# Challenges in the Heterologous Production of Furanocoumarins in *Escherichia coli*

**DOI:** 10.3390/molecules27217230

**Published:** 2022-10-25

**Authors:** Joana L. Rodrigues, Daniela Gomes, Lígia R. Rodrigues

**Affiliations:** 1 CEB—Centre of Biological Engineering, University of Minho, 4710-057 Braga, Portugal; 2 LABBELS—Associate Laboratory, 4710-057 Braga, Portugal

**Keywords:** coumarins biosynthesis, *Escherichia coli*, heterologous production, umbelliferone, scopoletin, esculetin, *p*-coumaroyl-CoA 2′-hydroxylase, prenyltransferase, marmesin synthase, psoralen synthase

## Abstract

Coumarins and furanocoumarins are plant secondary metabolites with known biological activities. As they are present in low amounts in plants, their heterologous production emerged as a more sustainable and efficient approach to plant extraction. Although coumarins biosynthesis has been positively established, furanocoumarin biosynthesis has been far more challenging. This study aims to evaluate if *Escherichia coli* could be a suitable host for furanocoumarin biosynthesis. The biosynthetic pathway for coumarins biosynthesis in *E. coli* was effectively constructed, leading to the production of umbelliferone, esculetin and scopoletin (128.7, 17.6, and 15.7 µM, respectively, from tyrosine). However, it was not possible to complete the pathway with the enzymes that ultimately lead to furanocoumarins production. Prenyltransferase, psoralen synthase, and marmesin synthase did not show any activity when expressed in *E. coli*. Several strategies were tested to improve the enzymes solubility and activity with no success, including removing potential *N*-terminal transit peptides and expression of cytochrome P450 reductases, chaperones and/or enzymes to increase dimethylallylpyrophosphate availability. Considering the results herein obtained, *E. coli* does not seem to be an appropriate host to express these enzymes. However, new alternative microbial enzymes may be a suitable option for reconstituting the furanocoumarins pathway in *E. coli*. Nevertheless, until further microbial enzymes are identified, *Saccharomyces cerevisiae* may be considered a preferred host as it has already been proven to successfully express some of these plant enzymes.

## 1. Introduction

**Figure 1:**
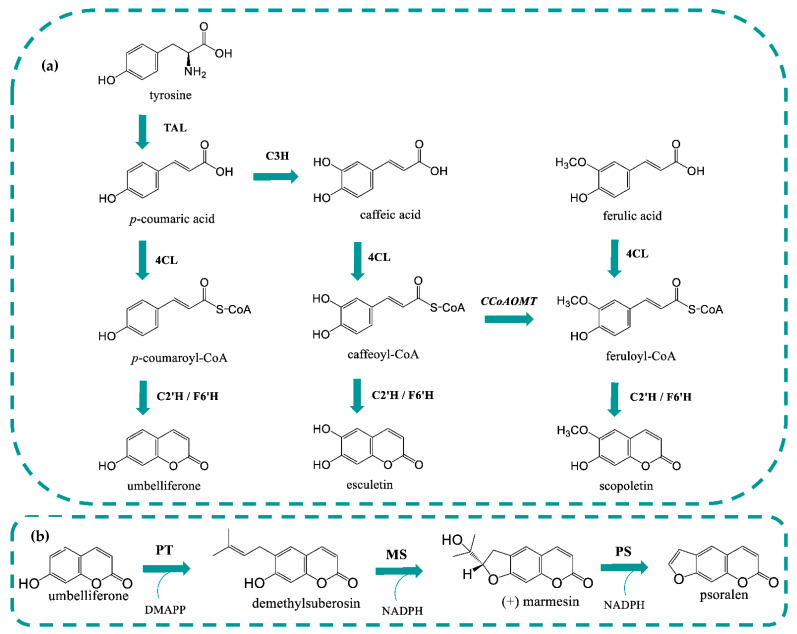
Coumarin’s biosynthetic pathway in *Escherichia coli* (**a**). Linear furanocoumarin’s pathway in plants (**b**). 4CL1: 4-coumarate-CoA ligase 1; C2′H: *p*-coumaroyl-CoA 2′-hydroxylase; C3H: 4-coumarate 3-hydroxylase; CCoAOMT: caffeoyl-CoA 3-*O*-methyltransferase; DMAPP: dimethylallylpyrophosphate; F6′H: feruloyl-CoA 6′-hydroxylase; MS: marmesin synthase; NADPH: nicotinamide adenine dinucleotide phosphate; PS: psoralen synthase; PT: prenyltransferase; TAL: tyrosine ammonia lyase.

Coumarins are phenolic compounds produced in plants as secondary metabolites. Most known coumarins include coumarin, umbelliferone, scopoletin and esculetin and have several interesting biological activities, including anti-inflammatory, anticancer, neuroprotective, antifungal, antibacterial, antidiabetic, antiepileptic, cardiovascular protective, antiulcerogenic, and antidiarrheal, among others [1,2,3,4,5,6,7,8,9]. For example, several coumarins have been studied in clinical trials and demonstrated potential in the treatment of leukemia, renal, and prostate cancers, among others [10,11,12]. In addition, the synthetic coumarin derivative warfarin is frequently prescribed as an anticoagulant for thrombosis treatment [13]. Umbelliferone derivatives such as furanocoumarins have also reported several therapeutic properties [14]. Furanocoumarins (frequently simply referred as psoralens), such as xanthotoxin and bergapten, have been used in clinics for several years to treat severe cases of psoriasis and vitiligo and as a first line of treatment of mycosis fungoides [15]. Currently, there are several pharmaceuticals on the market (e.g., Uvadex^®^, Oxsoralen^®^, 8-MOP^®^, and Melanocyl^®^) with these active principles approved by the EMA/FDA that are used in PUVA therapy consisting of the combination of psoralens with UVA radiation. Furanocoumarin’s potential is also being evaluated for treating cancer, graft-versus-host disease and in the prevention and treatment of solid organ transplantation rejection, among others [16,17,18]. In addition to their application as pharmaceuticals, several coumarin derivatives including furanocoumarins could be used as agrochemicals due to their phytotoxic, antibacterial, antifungal, insecticide, and herbicide properties [19,20,21]. Coumarins and their derivatives, as other plant secondary metabolites, are produced in very low amounts in plants. Therefore, the extraction process, besides being environmentally unfriendly, is also costly and ineffective [14]. Consequently, more efficient and sustainable production processes need to be developed. Heterologous production has been suggested as a potentially viable solution as it is not limited by plant availability, seasonality, or environmental factors and is a greener approach [22,23]. Additionally, metabolic engineering and synthetic biology tools allow the construction of more and more efficient strains capable of producing the heterologous value-added compounds with higher titers and yields [24,25]. This type of approach, although with its own challenges [14,26,27], has been successfully used to produce several compounds such as curcuminoids, flavonoids, and sesquiterpenoids produced in plants in low amounts [22,28,29]. Coumarins heterologous production has already been explored in *Escherichia coli* [30,31,32] and *Saccharomyces cerevisiae* [33]. The heterologous production of these compounds in microbes starts by the introduction of the phenylpropanoids pathway where tyrosine ammonia lyase (TAL) converts tyrosine to *p*-coumaric acid (Figure 1). Then, 4-coumarate-CoA ligase (4CL) converts *p*-coumaric acid to *p*-coumaroyl-CoA. This enzyme is also able to convert other hydroxycinnamic acids, such as ferulic and caffeic acid to the respective CoA esters. Finally, a 2-oxoglutarate-dependent dioxygenase, which can be a feruloyl-CoA 6′-hydroxylase (F6′H) or a *p*-coumaroyl-CoA 2′-hydroxylase (C2′H), depending on the substrate specificity, converts *p*-coumaric acid, caffeic acid and ferulic acid to umbelliferone, esculetin and scopoletin, respectively. So far, up to 356.59 mg/L of umbelliferone were obtained in a *E. coli* tyrosine overproducing strain [31]. The other coumarins have also been produced but in lower amounts compared to umbelliferone [30,32].

The heterologous production of coumarin derivatives, in particular of umbelliferone derivatives has been much less explored. As far as we know, furanocoumarins have never been produced in microbial hosts. Some of the steps have been individually tested but the pathway has never been fully assembled [14]. So far, there is only one preliminary study that shows that umbelliferone can be converted in *E. coli* to demethylsuberosin by a plant prenyltransferase (PT) and then to marmesin by a microbial marmesin synthase (MS) [34]. Previous reports demonstrated that PTs could not be expressed in *E. coli* [35], being usually evaluated in the plant *Nicotiana benthamiana* [35,36,37]. Additionally, MS and psoralen synthase (PS) characterization studies [38,39,40] have also suggested that *E. coli* is not a good host to express these enzymes. Therefore, these steps need to be investigated further to elucidate if the heterologous production of these compounds in *E. coli* can be considered a viable alternative.

Therefore, the main goal of this work was to evaluate the possibility of producing furanocoumarins in *E. coli* through the in-depth study of several steps of the pathway. In this work, the heterologous production of coumarins was established and used as a basis for the study of the next steps in the furanocoumarin’s pathway (PT, MS, and PS steps). The heterologous production of umbelliferone, esculetin and scopoletin was well succeeded, leading to 128.7, 17.6, and 15.7 µM, respectively, from tyrosine. However, it was not possible to complete the biosynthetic pathway with a PT enzyme to produce demethylsuberosin from tyrosine. The PT, MS, and PS enzymes from the furanocoumarin’s pathway tested did not demonstrate catalytic activity in vivo or in vitro. Strategies, such as the removal of potential *N*-terminal transit peptides and expression of a cytochrome P450 reductase, chaperones, and/or enzymes to increase the availability of dimethylallylpyrophosphate (DMAPP) have been attempted to improve the enzyme’s solubility and activity, although without success. Therefore, it was concluded that *E. coli* might not be a suitable host for the expression of this pathway using plant enzymes. Future research may focus in using microbial enzymes or other hosts such as *S. cerevisiae* possibly adopting co-culture/sequential culture approaches.

## 2. Results and Discussion

### 2.1. Heterologous Production of Umbelliferone using C2′H

To convert *p*-coumaroyl-CoA to umbelliferone, we selected the enzyme IbF6′H2-2-1 from Ipomoea batatas [41]. This isoenzyme previously demonstrated to have similar affinity to *p*-coumaroyl-CoA and feruloyl-CoA as substrates [41] when expressed in *E. coli* BL21 as it was verified for other isoenzymes. Since the F6′H nomenclature is normally used on enzymes with a higher F6′H activity than C2′H, we herein refer to this enzyme simply as C2′H.

**Figure 2:**
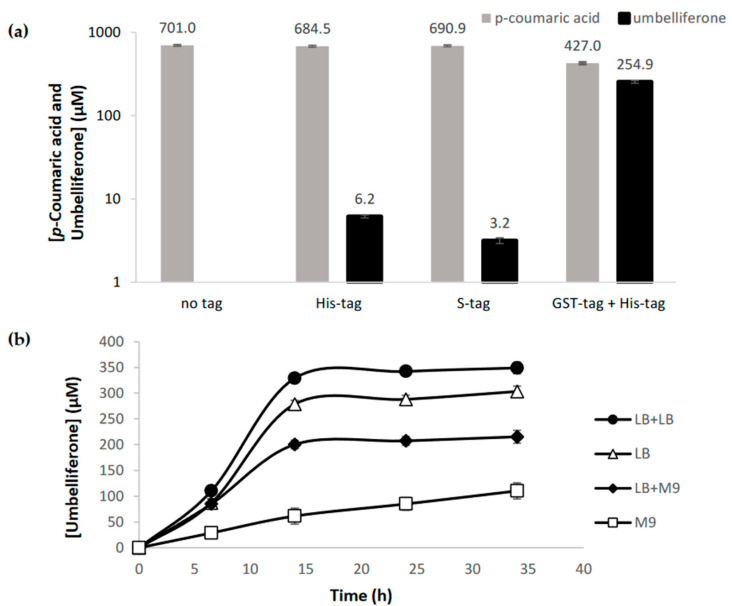
Umbelliferone production from *p*-coumaric acid using *p*-coumaroyl-CoA 2′-hydroxylase (C2′H) expressed with different tags (His-tag, S-tag, and GST-tag) (**a**) and using different culture media (LB + LB, LB, LB + M9, and M9) (**b**). *p*-Coumaric acid was added at a concentration of 0.7 mM (**a**) and 1 mM (**b**).

C2′H enzymes are produced in the cytoplasm of the plant whereby they do not have predictable signal sequences in the *N*- or *C*- terminals [42] as can be easily confirmed using different bioinformatic tools (LocTree3, iPSORT, SignalP, TargetP). However, it has been reported that C2′H/F6′H have low solubility when expressed in *E. coli*. Therefore, we tested the production of umbelliferone with C2′H not only in the commonly used high-copy plasmid pRSFDuet-1, but also in pET28GST-LIC that contains a glutathione S-transferase (GST)-tag. This tag has been previously used to increase F6′H stability and solubility [32]. Herein, C2′H cloned in different plasmids was combined with 4CL1 from Arabidopsis thaliana (pAC-4CL1) to produce umbelliferone from supplemented *p*-coumaric acid (0.7 mM). It was not possible to observe any production when C2′H was expressed in pRSFDuet-1 without any fusion tag, and the production was very residual when His or S-tags were used (Figure 2a). When C2′H was expressed with GST-tag, the umbelliferone production was significant demonstrating that this tag had a positive effect in C2′H expression although no significant differences were observed in the SDS-PAGE (sodium dodecyl sulfate polyacrylamide gel electrophoresis) gel (Figure S1). C2′H expressed in both plasmids was mostly in insoluble fraction.

The highest production herein obtained, 254.9 µM (41.33 mg/L), was higher than the one obtained by Lin et al. [30] (4.3 mg/L) that used a 4CL2 from *A. thaliana* and a F6′H from *I. batatas* (IbF6′H2-1-1) that also has a high specificity for *p*-coumaroyl-CoA [41]. However, it was considerably lower compared to the umbelliferone production (82.9 mg/L) obtained from *p*-coumaric acid in the Yang et al. [32] study. In this study, *Oryza sativa* 4CL and IbF6′H2-1-8 from *I. batatas* were used. IbF6′H2-1-8, according to in vitro studies, presents a higher catalytic efficiency and a higher preference for *p*-coumaroyl-CoA than feruloyl-CoA [41].

Previous studies reported the coumarins production in different culture media. The protein production phase is always performed in LB. However, in some cases in the coumarins production phase this LB is replaced by fresh LB, M9 (2% glucose) or YM9 (M9 2% glucose + 0.2% yeast extract) [30,31,32]. Therefore, we decided to also evaluate if the production phase or the complete experiment in M9 could be more favorable. Our previous studies in hydroxycinnamic acids and curcuminoids production demonstrated that M9 [43,44,45,46,47] could be an interesting option to produce these compounds. Preliminary studies (Figure 2a) were performed using LB in the protein production phase and then, fresh LB in the coumarin production phase, as it was demonstrated by Yang et al. [32] that fresh LB obtained more scopoletin than M9 or YM9. Our study also confirmed that fresh LB allows to produce more umbelliferone than fresh M9 or than using the same LB or M9 from the beginning to the end of the experiment (Figure 2b). In these assays, 1 mM of *p*-coumaric acid was used as precursor. The improved results with fresh LB compared to when the same LB is used for both production phases (protein and coumarins) makes sense as new nutrients are available, and the toxicity is lower as the by-products produced by *E. coli* during the protein production phase are removed. As expected, the production in only M9 minimal medium was very low since the bacterial growth is very slow in this medium and the bacteria takes more time to adapt.

### 2.2. C2′H substrate Specificity

**Figure 3:**
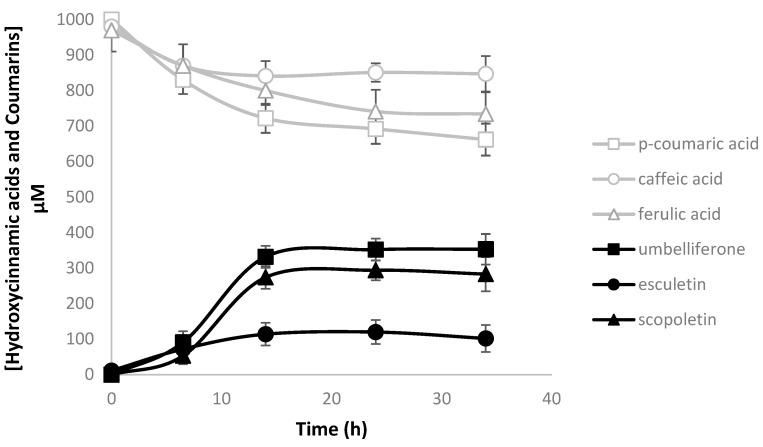
Coumarin production in *Escherichia coli* using different hydroxycinnamic acids as precursors. Umbelliferone, esculetin, and scopoletin are catalyzed from *p*-coumaric acid, caffeic acid and ferulic acid when pAC-4CL1 and pET28GST-C2′H are expressed. 4CL1: 4-coumarate-CoA ligase 1; C2′H: *p*-coumaroyl-CoA 2′-hydroxylase.

In order to confirm C2′H specificity, three different precursors were fed to the culture medium (LB + fresh LB): *p*-coumaric acid, caffeic acid, and ferulic acid (at 1 mM final concentration) that may be converted to umbelliferone, esculetin, and scopoletin, respectively (Figure 1). *E. coli* BL21 (DE3) carrying pAC-4CL1 and pET28GST-C2′H was able to produce the three different coumarins in different amounts (Figure 3). As expected, the production of umbelliferone and scopoletin was obtained in similar amounts, with umbelliferone being ~1.25-fold higher than scopoletin. Esculetin was also produced but in lower amounts, which is not in accordance with the literature [41] as it has been previously reported that IbF6′H2-2-1 (C2′H from this study) did not show any activity when caffeoyl-CoA was used as substrate in the in vitro reactions. It is possible that the activity was very low to be detected in the in vitro reactions. This was also verified by Yang et al. [32], who used two F6′H enzymes that produced esculetin in vivo and could not produce it in vitro [41]. Although the coumarins productions obtained in this study are lower than those reported by Yang et al. [32], thus suggesting that the enzyme from this study has lower activity towards the substrates, the proportion of coumarins obtained is very similar showing that they have a similar substrate specificity.

### 2.3. Heterologous Production of Coumarins from Tyrosine

**Figure 4:**
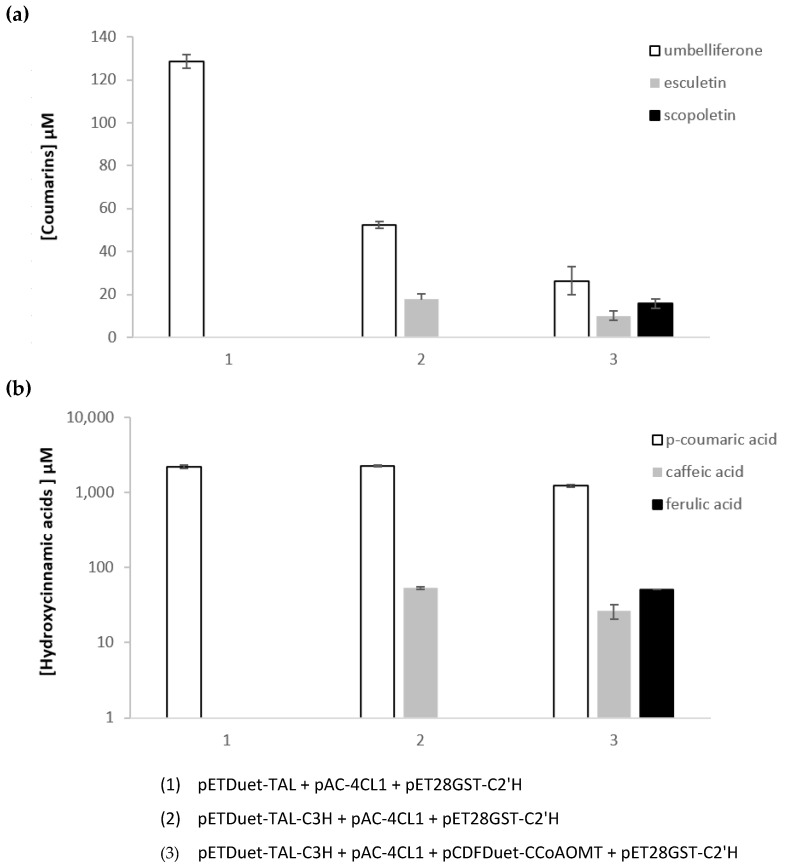
Coumarin production in *Escherichia coli* using tyrosine as precursor and different combinations of plasmids. Coumarins concentrations (**a**), hydroxycinammic acids concentrations (**b**). 4CL1: 4-coumarate-CoA ligase 1; C2′H: *p*-coumaroyl-CoA 2′-hydroxylase; C3H: 4-coumarate 3-hydroxylase; CCoAOMT: caffeoyl-CoA 3-O-methyltransferase; TAL: tyrosine ammonia lyase.

To produce coumarins from the amino acid tyrosine, TAL from *Rhodotorula glutinis*, 4-coumarate 3-hydroxylase (C3H) from *Saccharothrix espanaensis* and caffeoyl-CoA 3-O-methyltransferase (CCoAOMT) from *Medicago sativa* were used (Figure 1). All these genes were previously selected to construct caffeic acid and curcuminoids pathways by our research group [43,45,46]. TAL converts tyrosine to *p*-coumaric acid that is then converted to caffeic acid by C3H. 4CL1 is used to convert the hydroxycinnamic acids into their CoA esters and lastly, CCoAOMT converts caffeoyl-CoA to feruloyl-CoA. The combination of these genes allows to obtain all the three CoA esters (*p*-coumaroyl-CoA, caffeoyl-CoA, and feruloyl-CoA) that are finally converted to the respective coumarins by C2′H. In these experiments, tyrosine (3 mM) was used as a precursor. Depending on the combination of plasmids used, one, two, or three coumarins were produced (Figure 4). *p*-Coumaric acid was highly accumulated in all the experiments. This is consistent with previous results using TAL [43,44,45,46] as this enzyme is highly efficient. The other enzymes of the pathway are not as efficient. Nevertheless, it was possible to observe a small accumulation of caffeic and ferulic acid that justifies the low production of the respective coumarins. Umbelliferone was produced in higher amounts since its precursor is highly available. The production of scopoletin was relatively high taking into account the available amounts of ferulic acid, again demonstrating the high affinity of C2′H to feruloyl-CoA [41]. Comparing these results to other published studies, it is possible to conclude that the production of coumarins is in some cases lower which was expected as the production of coumarins directly from the hydroxycinnamic acids was already lower [32]. Then, the increase in the number of genes involved in the pathway also increased the metabolic burden leading to lower productions [44]. Nevertheless, the amount of umbelliferone produced was sufficient to continue our study and test the next step of the pathway catalyzed by a PT enzyme (Section 2.4).

### 2.4. The Prenyltransferase (PT) Step

The conversion of umbelliferone to demethylsuberosin catalyzed by a 6-PT is the entry point of the furanocoumarins pathway [14]. Plant PTs are known to be membrane-bound enzymes and, consequently, they are difficult to express in microorganisms. Therefore, its expression is not usually efficient in *E. coli*, with *N. benthamiana* often being used [35,36,37]. Recently, Bu et al. [34] showed that codon-optimized PT from *Petroselinum crispum* (*Pc*PT) and PT2 from *Pastinaca sativa* (*Ps*PT2) could be used to convert umbelliferone to demethylsuberosin and osthenol (angular furanocoumarin pathway), respectively, in *Streptomyces xiamenensis* and in *E. coli.* This was the first time that a plant PT enzyme was reported to be functional in a bacterium. Considering these results, we selected *Pc*PT (codon-optimized for *E. coli*) to convert umbelliferone to demethylsuberosin in vivo. *Pc*PT was cloned in pETDuet-TAL multiple cloning site 2 (MCS2) or in pCDFDuet-1 to be combined with the other plasmids (pAC-4CL1 and pET28GST-C2′H) and tyrosine was used as substrate. However, no demethylsuberosin production was observed. Therefore, *p*-coumaric acid and umbelliferone were also tested as precursors in vivo with no success. To rule out the idea that *Pc*PT was not being expressed in enough amounts due to its presence in a medium-copy plasmid (pETDuet-1/pCDFDuet-1), we attempted the *Pc*PT cloning in the high copy number pRSFDuet-1 plasmid and also in pET28GST-LIC to evaluate if GST-tag could improve the activity. *Pc*PT activity was evaluated in vitro using these plasmids. However, again demethylsuberosin was not detected. In addition to *Pc*PT, codon-optimized PT1 from *P. sativa* (*Ps*PT1) known to convert umbelliferone to demethylsuberosin [35] (although not as efficiently as *Pc*PT [36]) was also evaluated in vitro with no success. SDS-PAGE gels also did not reveal the desired proteins (data not shown) demonstrating that both PT enzymes were not being successfully or efficiently expressed in *E. coli*.

Plant PT enzymes, as membrane-bound proteins, contain transmembrane helices and are known to have transit peptides that guide them to transmembrane regions [48]. Therefore, to characterize *Citrus limon* PT using *S. cerevisiae* as a host, Munakata et al. [49] deleted 36 amino acids from the *N*-terminal region related to a transit peptide that was targeting the enzyme to the plastid. This strategy allowed them to successfully characterize the PT enzyme in yeast. It was suggested that the removal of the transit peptides can help the PT, the substrates, and the prenyl donors to be closer, improving the prenylation efficiency in microorganisms [48]. Hence, we decided to evaluate if this strategy could be used to improve the expression also in bacteria. We started by analyzing the PT sequences using several bioinformatic tools. The results were different depending on the tool used (Figures S2–S13). ChloroP (currently discontinued) did not predict any chloroplast transit peptide in *Pc*PT. This was not expected as Karamat et al. [36] mentioned that ChloroP predicted a transit peptide of 48 aa targeting chloroplast. However, LocTree3 and BaCelLo predicted that the protein subcellular localization was the chloroplast/chloroplast membrane. iPSORT predicted a mitochondrial targeting peptide at *N*-terminal while TargetP and SignalP did not predict a signal peptide (mitochondrial or chloroplast). Since these results were not coherent, it was decided to consider a transited peptide with 48 aa. Bioinformatic analysis of *P. sativa* PT1 also presented contradictory results. ChloroP predicted that *Ps*PT1 had a *N*-terminal chloroplast transit peptide with a length of 48 aa. LocTree3 and BaCelLo also pointed to a chloroplast transit peptide. However, iPSORT predicted a mitochondrial targeting peptide at *N*-terminal. TargetP also predicted a transfer peptide with a cleavage site at position 32–33 aa. However, SignalP-5.0 did not predict any signal peptide. Again, we decided to consider a transit peptide with 48 aa. Therefore, we deleted the first 144 bp from both genes. A second codon was added that codified alanine (Met-Ala) as it is a preferred second codon in *E. coli*, present in several highly expressed genes (e.g., *β*-galactosidase gene) [50]. GST was removed during the cloning in pET28GST-LIC to properly test the *N*-terminal modification. Unfortunately, the expression of truncated PTs did not show any improvement in in vivo and in vitro experiments, and in SDS-PAGE (data not shown).

Although the prenyl donor DMAPP availability was previously identified as a limiting step in the production of several aromatic compounds in microbes [48,51,52], we did not expect that it was so extreme that no production at all was observed. Indeed, Bu et al. [34] did not report any supplementation of DMAPP in the in vivo experiments with *E. coli*. Nevertheless, we repeated the in vivo experiments by expressing pMVA plasmid, containing several genes from the mevalonate (MVA) pathway responsible for the synthesis of DMAPP. The plasmid contained genes from *Enterococcus faecalis* (*mvaS*, hydroxymethylglutaryl-CoA synthase; *mvaE*, bifunctional acetoacetyl-CoA thiolase, and 3-hydroxy-3-methylglutaryl-CoA reductase), *E. coli* BL21 (*idi*, isopentenyl pyrophosphate—DMAPP isomerase) and *S. cerevisiae* (*erg12*, MVA kinase; *erg8*, phospho-MVA kinase; *erg19*, MVA pyrophosphate decarboxylase). Again, no improvement was observed in the in vivo experiments.

### 2.5. The Psoralen Synthase (PS) Step

PS is responsible for the conversion of marmesin to psoralen. This enzyme was first characterized in 2007 by Larbat et al. [38]. This enzyme belongs to cytochrome P450 (CYP450) family and is known to be difficult to express in *E. coli* as this organism does not have in its genome any CYP450 or CYP450 reductases (CPR) (redox partners to enhance CYP450 activity). Actually, preliminary tests in a previous work [38] suggest that PS enzyme from *Ammi majus* (CYP71AJ1) was not successfully expressed in *E. coli* and in *S. cerevisiae* without any protein modification. Nevertheless, in this study we selected PS enzyme from *A. majus* as from the few enzymes already characterized [38,39], it is the one that showed more activity towards marmesin. Codon-optimized PS was cloned in pRSFDuet-1 and pET28GST-LIC. The expression was evaluated in SDS-PAGE (Figure S14). No protein of interest was observed when expressed in pRSFDuet-1. In pET28GST-PS, it was possible to observe a protein highly expressed compared to the time 0 control. The expected protein band in this plasmid was expected around 82 kDa. However, the gels show 2 bands very close to each other around 70–60 kDa. The bands very close suggest that PS protein migrated as a doublet protein on SDS-PAGE gels. A small band around 30 kDa is also observed that can be related to proteolysis even though a protease inhibitor was used. The differences in size may be related to gel-shifting that is common [53] and apparently even more common for membrane proteins [54]. PS, as a CYP450, is a membrane-bound protein. Bioinformatic analysis using SignalP-5.0 showed a possible signal sequence of 30 aa and iPSORT predicted a mitochondrial targeting peptide. TMHMM-2.0 also predicted a transmembrane helix at 12–29 aa, which is in good agreement with the SignalP-5.0 prediction. LocTree3 predicts an endoplasmic reticulum localization. ChloroP did not predict any signal peptide, however, BaCelLo predicted a chloroplast localization which is contradictory. Bioinformatic results are presented in supplementary material (Figures S15–S21). Although some of the bioinformatic tools suggest a 29–30 aa signal sequence, Larbat et al. [38] considered the complete sequence of 37 aa preceding the highly conserved proline-rich region (37-PPSPP-43) known to link the *N*-terminal transmembrane sequence to the CYP450 catalytic domain [55]. Larbat et al. [38] replaced the 37 aa *N*-terminal for the CYP73A1 *N*-terminal in order for PS being successfully expressed in *S. cerevisiae*. Similar strategies have been used with success to express other CYP450 in yeast [35]. Additionally, this type of strategy has also been successfully used to express this type of enzymes in *E. coli* [56]. The truncation or replacement of CYP450 and CPR *N*-terminal regions led to an increase in CYP450 and CPR solubility and expression [57,58]. *N*-terminal has been frequently replaced by Met-Ala (as previously discussed) [58,59] or by a ‘universal’ *N*-terminal sequence (e.g., 8RP, MALLLAVF; 2C3, MAKKTSSKGK; 2B1, MAKKTSSKGKLPPG(PS)) [58,59,60,61,62,63]. More complex *N*-terminal sequences containing 84–214 aa have also been used (e.g., 28-tag, Sumo, MBP) [59,61].

After the literature review, it was decided to test the replacement of 37 aa *N*-terminal for MA (Met-Ala), 8RP, 2C3, and 28-tag (84 aa). The new *N*-terminal sequences were codon optimized for *E. coli*. Modified PS genes were cloned in pRSFDuet-1 and pET28GST-LIC. The SDS-PAGE results can be observed in Figures S22–S25. No PS protein was observed when MA *N*-terminal was used. However, high ∆37PS expression in insoluble phase was observed when the other three *N*-terminals were used, especially when cloned in pET28GST-LIC plasmid. Expression in pRSFDuet-1 was generally lower with exception of when 2C3 was used. In addition, when pRSFDuet-1 was used, the bands of interest were observed at the expected place (53–57 kDa). When expressed in pET28GST-LIC, the results were similar to the ones previously obtained before the *N*-terminal replacement (Figure S14), with a very intense band near the correct size, suggesting 2 bands very close to each other in the 8RP and 28tag cases, and one smaller band at around 30 kDa. We did not find any explanation for the presence of this lower band since in these cases the other band(s) had the correct size making the previously suggested proteolysis less probable.

As mentioned, *E. coli* does not contain a CPR enzyme. Therefore, CPR2 from *A. thaliana* [59], successfully used in the production of baicalein and scutellarein in *E. coli,* was selected for this work. This electron transfer partner was expressed in the supplied plasmid pETDuet-CPR2 in combination with other plasmids carrying PS variants (pET28GST-PS, pRSFDuet-PS, pET28-MA-∆37PS, pRSFDuet-MA-∆37PS, pET28–8RP-∆37PS, pRSFDuet-8RP-∆37PS, pET28-2C3-∆37PS, pRSFDuet-2C3-∆37PS, pET28-28tag-∆37PS, and pRSFDuet-28tag-∆37PS). The protein extracts were used in in vitro reactions to evaluate PS and PS variants activity. Activity of insoluble fractions was also tested as aggregation and inclusion body formation may result in only a moderate loss of activity [64]. Unfortunately, it was not possible to detect the product psoralen in any of the combinations tested.

PS protein was visible in the protein gels in very high amounts but in the insoluble phase. It is frequent that the overexpression of heterologous proteins using strong promoters can result in misfolding and aggregation of the heterologous proteins as inclusion bodies [65,66]. All this induces *E. coli* heat-shock response. However, this response is usually not enough for the high-speed production of difficult to fold proteins [65,66]. Therefore, the overexpression of proteins that assist in protein folding such as chaperones (e.g., GroEL/GroES) in combination with the protein(s) of interest has been used to improve protein folding, stability, and activity [67]. Therefore, GroEL and GroES chaperones were cloned in pCDFDuet-1, which later was combined with pETDuet-CPR2 and pET28GST/pRSFDuet-PS (and PS variants). The chaperones expression was evaluated in an SDS-PAGE gel (Figure S26). It is possible to observe that GroEL is highly expressed in both fractions. Since GroEL and PS variants are almost the same size (55–57 kDa) it is not possible to observe if the expression of PS variants improved in the soluble phase or not (Figures S26–S27). Afterwards, the PS variants’ activity was evaluated in vitro by expressing the enzymes with GroESL and CPR2. However, no psoralen was detected either using soluble or insoluble fractions. In the future, other strategies may be considered such as the refolding of purified inclusion bodies or the use of weaker promoters [66,68,69].

### 2.6. The Marmesin Synthase (MS) Step

The MS step was first described in 1988 by Hamerski and Matern [70] but only in 2021 was an enzyme able to perform this step discovered [40]. Villard et al. [40] identified an enzyme capable of converting demethylsuberosin to marmesin with very high affinity for the first time. This enzyme, CYP76F112, was identified in *Ficus carica* and also belongs to CYP450 family. The authors were able to successfully express and characterize this enzyme in *S. cerevisiae* with no modifications.

We started by analyzing the CYP76F112 protein sequence using bioinformatics tools. In the case of this MS protein, the bioinformatic results were in general very consistent (Figures S28–S34). iPSORT, TargetP and SignalP predicted that MS has a secretory signal peptide at its *N*-terminal with a cleavage site between position 25 and 26. LocTree3 pointed to a localization in the endoplasmic reticulum membrane and TMHMM also predicts a transmembrane helix at position 7 to 26. Considering these results, we decided to assess native CYP76F112 expression and a version without the first 25 aa and with 8RP *N*-terminal. The protein expression was evaluated using SDS-PAGE; howver, no protein was observed with any version, even when MS proteins were co-expressed with GroESL chaperones and CPR2 (data not shown). The in vitro reactions also did not show any production of marmesin.

## 3. Conclusions

As demonstrated in this work, producing furanocoumarins in *E. coli* is still a challenge. While coumarins have been successfully produced in this host in the past and also in this work, there was not enough information regarding the following steps of the pathway (PT, MS, and PS). In our study, despite several attempts it was not possible to confirm the PT, PS, or MS activity when the enzymes were expressed in *E. coli*. Therefore, new approaches are required. In the future, PTs from microbial sources might be a better option for use in biotechnological applications [71]. Contrarily to plant PTs, these microbial enzymes are soluble and present a very broad substrate promiscuity in vitro for prenyl acceptors. So far, several microbial aromatic PTs that are involved in the production of secondary metabolites in microbes have been identified and successfully expressed in *E. coli* [51,72,73,74,75]. Although these enzymes are not involved in the production of furanocoumarins, at least one [51] was shown to catalyze the prenylation of umbelliferone to 7-dimethylallyl-umbelliferone. These enzymes, whose activity towards furanocoumarins needs to be analyzed, and new ones that will soon be identified, are expected to present a more suitable approach for the reconstruction of the furanocoumarin’s pathway in *E. coli*. Actually, the same reasoning can be used regarding the MS step. This plant enzyme was poorly explored since it has only been recently identified [40]. So far, everything indicates that this enzyme, as PS, is not a suitable option to express in *E. coli*. Therefore, the recently identified gene XimD from *S. xiamenensis* [34] that was able to produce marmesin in vivo in *S. xiamenensis* and in *E. coli* might be a better option.

Although *E. coli* has been considered an alternative chassis to produce several plant secondary metabolites, other hosts might be more suitable to engineer the furanocoumarins pathway. The *S. cerevisiae* genetic toolbox is also highly developed [76] and, as an eukaryote, it can perform post-translational modifications and contains intracellular compartments similar to plants [22,77]. This makes it a more suitable host for the expression of plant enzymes, including CYP450. This host has been used to express several PS [38,39] and, recently, also MS [40]. Although it was reported that it may not be a suitable host to express PTs from the furanocoumarins pathway [35,36], it has been used to express other membrane-bound PTs [52], including through PT *N*-terminal modification [36]. This strategy using this host may, in the future, achieve better results than the ones obtained in this study with *E. coli*. In addition, *S. cerevisiae* has been recently used to produce coumarins using a biosynthetic pathway [33]. However, the productions obtained are very low compared to the ones from *E. coli*. Actually, since the first part of the pathway involving coumarin biosynthesis has been successfully implemented in *E. coli*, and *S. cerevisiae* appears as a more promising host for the expression of the other enzymes of the pathway, a co-culture or sequential culture strategy using both hosts might be considered in the future. Co-culture studies using both hosts for polyphenolic compound production have been reported [78]. This interesting strategy might be viable as umbelliferone is transported to the extracellular medium after production and it has been reported that *S. cerevisiae* is able to uptake coumarins [79].

In conclusion, this work comprised an in-depth study of several steps from furanocoumarins biosynthetic pathway and several limitations have been pointed out. Hence, in the future, to achieve an efficient production of furanocoumarins in heterologous microbial hosts, all the hypotheses herein discussed should be considered.

## 4. Materials and Methods

### 4.1. Strains, Plasmids, and Chemicals

*E. coli* NZY5α (NZYTech—MB00401) was used for cloning and plasmid propagation. *E. coli* BL21 (DE3) (NZYTech—MB006) was used for the expression of the heterologous pathway. All plasmids used in this study are described in Table 1. pETDuet-TAL, pETDuet-TAL-C3H, and pCDFDuet-CCoAOMT constructions were previously described [43,45]. pAC-4CL1 (Addgene #35947), pET28GST-LIC (Addgene #26101), pETDuet-CPR2, and pYeDP60-CYP76F112 were kindly provided by Dr. Claudia Schmidt-Dannert [80], Dr. Cheryl Arrowsmith, Dr. Yong Wang [59], and Dr. Alain Hehn [40], respectively. pMVA (Addgene #121149) [75] and pETDuet-*Pc*PT-XimD-XimE (Addgene #172654) [34] were a gift from Dr. Min-Juan Xu. C2′H, *Ps*PT1 and PS were codon-optimized using ATGenium algorithm and synthesized by NZYTech (Lisbon, Portugal) and provided in pNZY29 and pHTP0 plasmids. The DNA sequences of the codon-optimized C2′H, *Ps*PT1 and PS genes are presented in Supplementary Table S1.

Super optimal broth with catabolite repression (SOC), lysogeny broth (LB) Miller medium, isopropyl β-D-1-thiogalactopyranoside (IPTG), and 5-bromo-4-chloro-3-indolyl-β-D-galactopyranoside (X-Gal) were purchased from NZYTech. *p*-Coumaric acid, caffeic acid and tyrosine were obtained from Sigma-Aldrich; ferulic acid, umbelliferone and scopoletin from Acros; esculetin and psoralen from Alfa Aesar; and demethylsuberosin and marmesin from Chengdu Biopurify Phytochemicals (Sichuan, China). Glucose (Acros), NH_4_Cl, NaCl, CaCO_3_ (Panreac), Na_2_HPO_4_ (Chem-Lab, Zedelgem, Belgium), MgSO_4_, CoCl_2_, FeCl_3_, CuCl_2_, ZnCl_2_, nicotinic acid, KH_2_PO_4_ (Riel-deHaën), thiamine (Thermo Fisher Scientific, Loughborough, United Kingdom), NaMoO_4_, pyridoxine, H_2_BO_3_, folic acid, biotin (Merck, Kenilworth, New Jersey, USA), riboflavin, and pantothenic acid (Sigma Aldrich) were used to prepare the M9 minimal medium. Ampicillin (VWR), chloramphenicol, kanamycin (NZYTech), spectinomycin (Alfa Aesar, Kandel, Germany), and sucrose (Labkem) were used for strain selection. Acetonitrile, trifluoroacetic acid and ethyl acetate were purchased from Fisher-Scientific. DMAPP (Merck), dithiothreitol (DTT), Tris (Fisher BioReagents, Fair Lawn, New Jersey, USA), NaH_2_PO_4_ (Scharlau), protease inhibitor (NZYTech), nicotinamide adenine dinucleotide phosphate (NADPH) (Panreac, Barcelona, Spain), and MgCl_2_ (VWR) were supplemented in enzymatic assays.

### 4.2. Construction of Plasmids

C2′H was amplified from pNZY29-C2′H and cloned in pRSFDuet-1 and pET28GST plasmids. *Pc*PT and ∆48*Pc*PT were amplified from pETDuet-*Pc*PT-XimD-XimE. *Ps*PT1 and ∆48*Ps*PT1 were amplified from pHTP0-*Ps*PT1. *Pc*PT was cloned in pETDuet-TAL, pCDFDuet-1, pRSFDuet-1 and pET28GST-LIC. PS and PS variants were amplified from pHTP0-PS. *Ps*PT1, ∆48*Pc*PT, ∆48*Ps*PT1, PS, MA-*∆37*PS, 8RP-*∆37*PS, 2C3-*∆37*PS, and 28tag-*∆37*PS were cloned in pRSFDuet-1 and pET28GST-LIC. The GroESL operon was amplified from an *E. coli* BL21 colony using colony-PCR. Afterwards, the chaperones were cloned as an operon in pCDFDuet-1 MCS1. CYP76F112 and 8RP-∆25CYP76F112 were amplified from pYeDP60-CYP76F112 and cloned in pRSFDuet-1. All the primers (Metabion/Eurofins) used in this study are presented in Supplementary Table S2.

Plasmid DNA was isolated using NucleoSpin Plasmid Miniprep Kit (Macherey-Nagel, Düren, Germany). The genes were amplified using Phusion High Fidelity DNA Polymerase (Thermo Fisher Scientific) and purified from agarose using NucleoSpin Gel and PCR Clean-up Kit (Macherey-Nagel). DNA was quantified using NanoDrop One (Thermo) and digested with the appropriate restriction enzymes (Supplementary Table S2). After digestion, DNA was purified using NucleoSpin Gel and PCR Clean-up Kit. T4 DNA ligase (Thermo) was used for ligation. Chemical transformation was performed using *E. coli* NZY5α competent cells. The constructed plasmids were verified by colony-PCR (Speedy supreme NZYTaq 2x Green Master Mix, NZYTech), digestion and sequencing (Eurofins). After confirmation, the plasmids were transformed in *E. coli* BL21 (DE3) competent cells. All enzymes and kits were used according to the manufacturer’s instructions.

### 4.3. Coumarins/Furanocoumarins Production

LB culture medium was used for plasmid propagation in *E. coli* BL21 (DE3) and for inoculums (200 rpm, 37 °C). For coumarin production, cultures were grown at 37 °C in 50 mL LB (250 mL flasks) from an optical density at 600 nm (OD_600_) of 0.1 to 0.6. Afterwards, the heterologous protein expression was induced with IPTG (0.1 mM final concentration), and the culture was incubated at 26 °C for 5 h. Then, the cells were collected by centrifugation and suspended in coumarin production medium (LB or M9 minimal medium). In two cases, cells were maintained in the same LB used for protein production or grew from the beginning in M9. IPTG (0.1 mM final concentration), precursors (*p*-coumaric acid, 0.7 mM–1 mM; caffeic acid, 1 mM; ferulic acid, 1 mM; or tyrosine, 3 mM) and antibiotics (ampicillin, 100 µg/mL; kanamycin, 50 µg/mL; spectinomycin, 100 µg/mL; and/or chloramphenicol, 25 µg/mL) were supplemented at time 0 depending on the specific experiment. M9 minimal medium contained (per L): 40 g glucose, 3 g KH_2_PO_4_, 6 g Na_2_HPO_4_, 0.5 g NaCl, 1 g NH_4_Cl, 110 mg MgSO_4_, 15 mg CaCl_2_, 340 mg thiamine and 5 g CaCO_3_. Vitamins (12.2 mg nicotinic acid, 10.8 mg pantothenic acid, 2.8 mg pyridoxine, 0.84 mg riboflavin, 0.12 mg biotin, and 0.084 mg folic acid) and trace elements (54 mg FeCl_3_, 4 mg NaMoO_4_, 4 mg ZnCl_2_, 4 mg CoCl_2_, 2 mg CuCl_2_, and 1 mg H_2_BO_3_) were also supplemented to M9.

Assays targeting demethylsuberosin production in vivo using PT enzymes were performed as coumarins production assays.

### 4.4. Coumarin/Furanocoumarin Extraction

The whole broth was extracted as coumarins can be detected in significant amounts in the supernatant and inside the cells. Samples (2 mL whole broth) were mixed with 2 mL of ethyl acetate, vortexed and centrifuged (15,000× *g*, 5 min). The supernatant was concentrated by solvent evaporation, resuspended in 200 µL of acetonitrile and analyzed by ultra-high-performance liquid chromatography (UHPLC). The same method was used to evaluate demethylsuberosin production in vivo, but a higher volume of whole broth samples was extracted.

### 4.5. UHPLC Analysis

Hydroxycinnamic acids (*p*-coumaric acid, caffeic acid, and ferulic acid), coumarins (umbelliferone, esculetin, and scopoletin) and furanocoumarins (demethylsuberosin, marmesin, and psoralen) were quantified by UHPLC. Supernatant was used to quantify the hydroxycinnamic acids while coumarins and furanocoumarins were extracted as described in 4.4. Samples from in vitro reactions were directly analyzed without any extraction.

Quantification was performed using the Shimadzu Nexera-X2 system (Shimadzu Corporation, Kyoto, Japan) (LC-30AD pump unit, DGU-20A 5R degasser unit, CTO-20AC column oven, CBM-20A system controller, SPD-M20A detector unit, SIL-30AC autosampler unit) and a Kinetex 2.6 µm Polar C18 100 Å LC column (150 mm × 4.6 mm) (Phenomenex, Alcobendas, Spain). Water with 0.1% (*v*/*v*) of formic acid and pure acetonitrile were used as mobile phase A and B, respectively. The following gradient was used at a flow rate of 1 mL/min for the determination of hydroxycinnamic acids, coumarins and furanocoumarins: 10–80% mobile phase B for 16 min, 80–10% for 1 min, and 10% mobile phase B for an additional 3 min. *p*-Coumaric acid, caffeic acid, and ferulic acid were detected at 310 nm at the retention time of 6.4, 5.1 and 7.7 min, respectively. Umbelliferone, esculetin and scopoletin were detected at 324, 340, and 342 nm and at a retention time of 6.6, 4.9, and 7.5 min, respectively. Finally, demethylsuberosin, marmesin and psoralen were detected at 334, 335, and 294 nm and at a retention time of 12.5, 8.3 and 10.0 min, respectively.

### 4.6. Protein Analysis

*E. coli* BL21 (DE3) cells carrying the plasmids of interest were grown in LB at 37 °C to an OD_600_ of 0.6. Then, IPTG was added at a final concentration of 1 mM to induce protein expression, the temperature was decreased to 26 °C and the culture was incubated for 6 h. Samples (10 mL) were taken at 0 and 6 h of induction and centrifuged. The pellets were resuspended in 1 mL of Tris-HCl buffer (10 mM, pH 7.8) supplemented with protease inhibitor (NZYTech) and lysed by sonication on ice (35% amplitude, 3 s ON plus 9 s OFF for a total of 5 min ON) using a microtip probe linked to a Vibra-cell processor (Sonics). After sonication, samples were centrifuged and resuspended, and the protein of the soluble and insoluble fractions was quantified using the Bradford reagent (Panreac). The expression levels were evaluated through SDS-PAGE (4% stacking gel and 10% running gel). Soluble and insoluble protein fractions were mixed with 2 x sample buffer (65.8 mM Tris–HCl pH 6.8, 26.3% glycerol, 0.01% bromophenol blue, 2.1% SDS, 5% β-mercaptoethanol) and denaturated at 95 °C in the heating block for 5 min. The protein ladders used were NZYColour Protein Marker II (NZYTech) and Blue Prestained Protein Standard—Broad Range (NEB). The gel was stained with Coomassie Blue R-250 for 15 min and de-stained using distilled water.

### 4.7. Enzymatic Assays

The protein extracts were obtained and quantified as described in Section 4.6. Afterwards, soluble and insoluble fractions were used to evaluate PT (Section 4.7.1), PS (Section 4.7.2) and MS (Section 4.7.3) activity in vitro. The reactions occurred at 27 or 30 °C with or without agitation for 4 h. Samples were taken at times 0, 2, and 4 h and analyzed as described in Section 4.5.

#### 4.7.1. PT In Vitro Reactions

PT enzymatic assays were performed based on [35,36,49]. One milliliter reactions were prepared in 100 mM Tris-HCl pH 8 (supplemented with protease inhibitor) containing 200 µM DMAPP, 200 µM umbelliferone, 10 mM MgCl_2_, and 1, 10, or 100 µg protein extract (soluble or insoluble phase). A quantity of 1 mM DTT was added in some tests.

#### 4.7.2. PS In Vitro Reactions

PS enzymatic assays were performed based on [38,39]. One milliliter reactions were prepared in 100 mM sodium phosphate pH 7 (supplemented with protease inhibitor) containing 1 mM NADPH, 0.1 mM marmesin and 1, 10, or 100 µg protein extract (soluble or insoluble phase).

#### 4.7.3. MS In Vitro Reactions

MS enzymatic assays were performed based on [40,70]. One milliliter reactions were prepared in 100 mM sodium phosphate pH 7 (supplemented with protease inhibitor) containing 200 µM NADPH, 50 µM demethylsuberosin, and 1, 10, or 100 µg protein extract (soluble or insoluble phase).

### 4.8. Bioinformatics

Enzymes transit peptides and subcellular localization were predicted using ChloroP (https://services.healthtech.dtu.dk/service.php?ChloroP-1.1) (discontinued, accessed on 1 May 2020), iPSORT (http://ipsort.hgc.jp/) [81] (accessed on 1 May 2020), SignalP-5.0 (https://services.healthtech.dtu.dk/service.php?SignalP-5.0) [82] (accessed on 1 May 2020), TargetP-2.0 (https://services.healthtech.dtu.dk/service.php?TargetP-2.0) [83] (accessed on 1 May 2020), TMHMM-2.0 (https://services.healthtech.dtu.dk/service.php?TMHMM-2.0) [84] (accessed on 1 May 2020), Loctree3 (https://rostlab.org/services/loctree3/) [85] (accessed on 1 May 2020), and BaCelLo (http://gpcr.biocomp.unibo.it/bacello/) [86] (accessed from 1 May 2020 to 1 June 2022).

## Figures and Tables

**Table 1:** Plasmids used in this study.

Plasmids	Construct	Source
pRSFDuet-1	RSF1030 *ori*, *lacI*, double P*_T7lac_*, Kan^R^	Novagen
pCDFDuet-1	CloDF13 *ori*, *lacI*, double P*_T7__lac_*, Spec^R^	Novagen
pNZY29-C2′H	ColE1(pBR322) *ori*, Amp^R^; pNZY29 carrying codon-optimized *p*-coumaroyl-CoA 2′-hydroxylase (C2′H) from *Ipomoea batatas*	NZYTech
pHTP0-*Ps*PT1	ColE1(pBR322) *ori*, Amp^R^; pHTP0 carrying codon-optimized prenyltransferase 1 from *Pastinaca sativa* (*Ps*PT1)	NZYTech
pHTP0-PS	ColE1(pBR322) *ori*, Amp^R^; pHTP0 carrying codon-optimized psoralen synthase (PS) from *Ammi majus*	NZYTech
pET28GST-LIC	ColE1(pBR322) *ori*, *lacI*, P*_T7lac_*, Kan^R^; carrying levansucrase (SacB) from *Bacillus subtilis* and glutathione S-transferase (GST)	Addgene (#26101)
pAC-4CL1	p15A *ori*, P*_lac_*, Cm^R^, pACYC184-derived plasmid carrying 4-coumarate-CoA ligase 1 (4CL1) from *Arabidopsis thaliana*	Addgene (#35947)
pETDuet-*Pc*PT-XimD-XimE	pBR322 *ori*, *lacI*, double P*_T7__lac_*, Amp^R^; pETDuet-1 carrying codon-optimized prenyltransferase from *Petroselinum crispum* (*Pc*PT) and marmesin synthase (XimD) and snoaL-like cyclase (XimE) from *Streptomyces xiamenensis*	Addgene (#172654)
pMVA	pCDFDuet-1 carrying hydroxymethylglutaryl-CoA synthase (MvaS) and bifunctional acetoacetyl-CoA thiolase and 3-hydroxy-3-methylglutaryl-CoA reductase (MvaE) from *Enterococcus faecalis,* isopentenyl pyrophosphate—dimethylallylpyrophosphate isomerase (IDI) from *E. coli* BL21 and mevalonate (MVA) kinase (ERG12), phospho-MVA kinase (ERG8) and MVA pyrophosphate decarboxylase (ERG19) from *Saccharomyces cerevisiae*	Addgene (#121149)
pETDuet-CPR2	pBR322 *ori*, *lacI*, double P*_T7__lac_*, Amp^R^; pETDuet-1 carrying cytochrome P450 reductase 2 (CPR2) from *A. thaliana*	[59]
pYeDP60-CYP76F112	pUC *ori*, 2 *µ ori*, *URA3*, Tet^R^, Amp^R^; pYeDP60 carrying marmesin synthase from *Ficus carica* (CYP76F112)	[40]
pETDuet-TAL	pBR322 *ori*, *lacI*, double P*_T7__lac_*, Amp^R^; pETDuet-1 carrying tyrosine ammonia lyase (TAL) from *Rhodotorula glutinis*	[45]
pETDuet-TAL-C3H	pETDuet-TAL carrying 4-coumarate 3-hydroxylase (C3H) from *Saccharothrix espanaensis*	[45]
pCDFDuet-CCoAOMT	pCDFDuet-1 carrying caffeoyl-CoA 3-*O*-methyltransferase (CCoAOMT) from *Medicago sativa*	[43]
pET28GST-C2′H	pET28GST-LIC without SacB and carrying C2′H in frame with GST and His-tag	This study
pRSFDuet-C2′H	pRSFduet-1 carrying C2′H without His-tag or S-tag in frame	This study
pRSFDuet-His-tag-C2′H	pRSFDuet-1 carrying C2′H with His-tag in frame	This study
pRSFDuet-C2′H-S-tag	pRSFDuet-1 carrying C2′H with S-tag in frame	This study
pETDuet-TAL-*Pc*PT	pETDuet-TAL carrying *Pc*PT	This study
pCDFDuet-*Pc*PT	pCDFDuet-1 carrying *Pc*PT	This study
pRSFDuet-*Pc*PT	pRSFDuet-1 carrying *Pc*PT	This study
pET28GST-*Pc*PT	pET28GST-LIC without SacB and carrying *Pc*PT	This study
pRSFDuet-∆48*Pc*PT	pRSFDuet-1 carrying truncated *Pc*PT	This study
pET28-∆48*Pc*PT	pET28GST-LIC without SacB and carrying truncated *Pc*PT	This study
pRSFDuet-*Ps*PT1	pRSFDuet-1 carrying *Ps*PT1	This study
pET28GST-*Ps*PT1	pET28GST-LIC without SacB and carrying *Ps*PT1	This study
pRSFDuet-∆48*Ps*PT1	pRSFDuet-1 carrying truncated *Ps*PT1	This study
pET28-∆48*Ps*PT1	pET28GST-LIC without SacB and carrying truncated *Ps*PT1	This study
pRSFDuet-PS	pRSFDuet-1 carrying PS	This study
pET28GST-PS	pET28GST-LIC without SacB and carrying PS	This study
pRSFDuet-MA-∆37PS	pRSFDuet-1 carrying MA-∆37PS	This study
pET28-MA-∆37PS	pET28GST-LIC without SacB and GST and carrying MA-∆37PS	This study
pRSFDuet-8RP-∆37PS	pRSFDuet-1 carrying 8RP-∆37PS	This study
pET28-8RP-∆37PS	pET28GST-LIC without SacB and GST and carrying 8RP-∆37PS	This study
pRSFDuet-2C3-∆37PS	pRSFDuet-1 carrying 2C3-∆37PS	This study
pET28-2C3-∆37PS	pET28GST-LIC without SacB and GST and carrying 2C3-∆37PS	This study
pRSFDuet-28tag-∆37PS	pRSFDuet-1 carrying 28tag-∆37PS	This study
pET28-28tag-∆37PS	pET28GST-LIC without SacB and GST and carrying 28tag-∆37PS	This study
pCDFDuet-GroESL	pCDFDuet-1 carrying chaperones GroES and GroEL from *E. coli* BL21 in an operon	This study

## Supplementary Materials

The following supporting information can be downloaded at: https://www.mdpi.com/article/10.3390/molecules27217230/s1, Table S1: Gene sequence of *p*-coumaroyl-CoA 2′-hydroxylase (C2′H), prenyltransferase 1 (PT1) and psoralen synthase (PS) with codon optimization; Table S2: Primers for PCR amplification of the genes of the biosynthetic pathway (forward (FW) and reverse (REV) primers) and sequencing; Figure S1: Protein SDS gels showing *p*-coumaroyl-CoA 2′-hydroxylase (C2′H) expression using different plasmids (pRSFDuet-C2′H and pET28GST-C2′H) at time zero (t0) of induction and after 6 h (t6) of induction. C2′H is expected around 40.41 kDa and GST + C2′H at 67.65 kDa. Arrows indicate where it is possible to observe the bands of interest. M: marker (NZYColour Protein Marker II—NZYTech); Figure S2: ChloroP results for PT from *Petroselinum crispum*; Figure S3: LocTree3 results for PT from *Petroselinum crispum*; Figure S4: BaCelLo results for PT from *Petroselinum crispum*; Figure S5: iPSORT results for PT from *Petroselinum crispum*; Figure S6: TargetP-2.0 results for PT from *Petroselinum crispum*; Figure S7: SignalP-5.0 results for PT from *Petroselinum crispum*; Figure S8: ChloroP results for PT1 from *Pastinaca sativa*; Figure S9: LocTree3 results for PT1 from *Pastinaca sativa*; Figure S10: BaCelLo results for PT1 from *Pastinaca sativa*; Figure S11: iPSORT results for PT1 from *Pastinaca sativa*; Figure S12: TargetP results for PT1 from *Pastinaca sativa*; Figure S13: SignalP-5.0 results for PT1 from *Pastinaca sativa*; Figure S14: Protein SDS gels showing psoralen synthase (PS) expression using different plasmids (pRSFDuet-PS and pET28GST-PS) at time zero (t0) of induction and after 6 h (t6) of induction. PS is expected around 55.9 kDa and GST+PS at 82.08 kDa. Arrows indicate where it is possible to observe the bands of interest. M: marker (NZYColour Protein Marker II—NZYTech); Figure S15: ChloroP results for PS from *Ammi majus*; Figure S16: iPSORT results for PS from *Ammi majus*; Figure S17: TMHMM-2.0 results for PS from *Ammi majus*; Figure S18: LocTree3 results for PS from *Ammi majus*; Figure S19: BaCelLo results for PS from *Ammi majus*; Figure S20: TargetP-2.0 results for PS from *Ammi majus*; Figure S21: SignalP-5.0 results for PS from *Ammi majus*; Figure S22: Protein SDS gels showing ∆37 aa psoralen synthase (PS) expression with MA N-terminal using different plasmids (pRSFDuet-MA-∆37PS and pET28-MA-∆37PS) at time zero (t0) of induction and after 6 h (t6) of induction. PS is expected around 52–54 kDa. M: marker (Blue Prestained Protein Standard, Broad Range—NEB); Figure S23: Protein SDS gels showing ∆37 aa psoralen synthase (PS) expression with 8RP N-terminal using different plasmids (pRSFDuet-8RP-∆37PS and pET28-8RP-∆37PS) at time zero (t0) of induction and after 6 h (t6) of induction. PS is expected around 53–55 kDa. Arrows indicate where it is possible to observe the bands of interest. M: marker (Blue Prestained Protein Standard, Broad Range—NEB); Figure S24: Protein SDS gels showing ∆37 aa psoralen synthase (PS) expression with 2C3 N-terminal using different plasmids (pRSFDuet-2C3-∆37PS and pET28-2C3-∆37PS) at time zero (t0) of induction and after 6 h (t6) of induction. PS is expected around 53–55 kDa. Arrows indicate where it is possible to observe the bands of interest. M: marker (Blue Prestained Protein Standard, Broad Range—NEB); Figure S25: Protein SDS gels showing ∆37 aa psoralen synthase (PS) expression with 28tag N-terminal using different plasmids (pRSFDuet-28tag-∆37PS and pET28-28tag-∆37PS) at time zero (t0) of induction and after 6 h (t6) of induction. PS is expected around 55–57 kDa. Arrows indicate where it is possible to observe the bands of interest. M: marker (NZYColour Protein Marker II—NZYTech); Figure S26: Protein SDS gels showing GroESL expression alone and when combined with cytochrome P450 reductase (CPR2) and 8RP-∆37PS at time zero (t0) of induction and after 6 h (t6) of induction. GroEL, GroES, CPR2 and 8RP-∆37PS are expected at round 57, 10, 79, and 55 kDa, respectively. Arrows indicate where it is possible to observe the bands of interest. M: marker (NZYColour Protein Marker II—NZYTech); Figure S27: Protein SDS gels showing GroESL expression combined with cytochrome P450 reductase (CPR2) and 2C3-∆37PS or 28tag-∆37PS at time zero (t0) of induction and after 6 h (t6) of induction. GroEL, GroES, CPR2, 2C3-∆37PS, and 28tag-∆37PS are expected at round 57, 10, 79, 55, and 56 kDa, respectively. Arrows indicate where it is possible to observe the bands of interest. M: marker (NZYColour Protein Marker II—NZYTech); Figure S28: ChloroP results for MS from *Ficus carica*; Figure S29: LocTree3 results for MS from *Ficus carica*; Figure S30: BaCelLo results for MS from *Ficus carica*; Figure S31: iPSORT results for MS from *Ficus carica*; Figure S32: TargetP-2.0 results for MS from *Ficus carica*; Figure S33: SignalP-5.0 results for MS from *Ficus carica*; Figure S34: TMHMM-2.0 results for MS from *Ficus carica.*
