# Supplementary material for: Challenges in the Heterologous Production of Furanocoumarins in Escherichia coli
Source: Molecules. 2022 Oct 25;27(21):7230. doi: 10.3390/molecules27217230 (PMC9656933; doi:10.3390/molecules27217230)
Supplement: Supplementary file 1 [file molecules-27-07230-s001.zip › molecules-1929143-supplementary.pdf]

molecules

MDPI

Supplementary material

Article

Challenges in the heterologous production of furanocoumarins in *Escherichia coli*

Joana L Rodrigues [1,2*], Daniela Gomes [1,2] and Lígia R Rodrigues [1,2]

1 CEB – Centre of Biological Engineering, University of Minho, 4710-057 Braga, Portugal; daniela.gomes@ceb.uminho.pt (DG); lrmr@deb.uminho.pt (LRR)
2 LABBELS – Associate Laboratory, Braga/Guimarães, Portugal.
* Correspondence: joanarodrigues@ceb.uminho.pt; joana.joanalucia@gmail.com (J.L.R); Tel.: +351-253604423

Table S1. Gene sequence of *p*-coumaroyl-CoA 2′-hydroxylase (C2′H), prenyltransferase 1 (PT1) and psoralen synthase (PS) with codon optimization.

Gene	Sequence
C2′H<br>(*Ipomoea batatas*)	ATGCCGTCGACCACTCTGAGCACTGTTCTTAGTGATATTAATGACTTCGTCGTGAAAC<br>AAGGCCACGGTGTTAAAGGCCTGTCCGAGCTGGGCTTACAAACCTTGCCGAACCAG<br>TATGTGCATCCGCCAGAGGAGCGTTTAAGCAGTATGGATGTGGTTTCCGATGACAGC<br>ATCCCGGTGATCGATGTCAGCAATTGGGAAGACCCGAAAGTGGCCAAATTGATCTG<br>CGACGCAGCCGAAAAGCGCGGTTTCTTTCAGATTGTGAATCATGGCATTCCGATTGA<br>AATGCTGGAGAAAGCAAAGGCCGCGACCTACCGTTTTTTTCGTGAACCAGCGGAGG<br>AAAAGAAAAAGTACAGCAAGGAAAACTGTCCAACTTCCCACGTGCGCTACAGTACT<br>AGTTTTCTTCCGCAGATTGAGAAGGCGCTGGAGTGGAAAGATCACCTGTCCATGTTT<br>TATGTGTCGGATGAGGAAGCGGCCCAGTACTGGCCGCCGTCGTGTCGCGATGATGCG<br>GTTGAATACCTGAAATCGTGTGAAATGGTTAGTCGTAAACTGTTAGAAGCACTTATG<br>CAGGGTCTGAACGTTAATCAGATTGACGATTCCAAGGAGAGTCTGTTAATGGGCAGT<br>CGTCGCATTAATATTAACTATTATCCAAAATGCCCGAACCCAGATTTGACCGTTGGT<br>GTGGGTCGCCATAGCGATATCAGCACCCTGACCCTGCTTTTACAGGACGACATCGGC<br>GGTTTATATGTTCGTAAGCTGGAACATGAGGCCTGGTCCCATGTGCCGCCGGTGAAA<br>GGTGCGTTAGTCATTAATATCGGCGATGCACTCCAGATTATGTCCAACGGTCGCTAT<br>AAAAGTATTGAACACCGCGTGATGGCGAACGAAAGTAATGATCGTATCTCGGTTCC<br>GGTGTTTGTGAACCCGCGCCCGAATGATATTGTGGGCCCGCTGCCGGAGGTTTTGGC<br>GTCGGGCGAAAAGCCAGTTTATAAACCAGTGCTGTATTCCGACTACGCAAAGCATTT<br>TTACCGTAAAGCGCATAACGGCAAAGATACGATCGCATTCGCGCGCATTGAATAA
PT1<br>(*Pastinaca sativa*)	ATGGCGCAGACCATTATGCACAGCCGCTTGAGCTCCGGTTTCCTCCATCTGCAGCGTG<br>ACAAGGGTTTTCGCACCCTGCCAACTCAGCGTCGTCATGCCAAAGTTGTTAACGGCG<br>ATCAAGAATTCGCGTTTCGTGTGGTGAGCTGCGATAAAAATTTAGACTCCACTAAAA<br>ATTTTAGTGGCAGCTGTGAAAAACCGATTCGCACCCATACTAATAAACTGCTGCAAA<br>CCATCAGCGCAACCAGCGATCGTGAAGCCATCATCCAACCGAAGGATGATTATGAA<br>GCCCCGTGGCAAAATACGCTGCGTCGTAAATGGGATGCGTTCTGTACGTTTGGCCGT<br>CCGTATTCCGCGATTTGTACGATTATCGGCATTAGCTCGGTGAGTCTGTTACCATTGA<br>CTTCGGTTAAAGACTTCAGCGCACCATATTTTGTCGGCTTGCTGCAGGCCCTGATTCC<br>ATTCCTGTGCGCCAATATTTACACCAGCGGCATCAATCAGCTGGTCGATGTGGACAT<br>CGACAAAATTAATAAGCCATACCTGCCGTTGGTTTCGGGTGAGTTTAGTCTGGGTGA<br>AGGCCGCGCCATTGTGTCGGCCCTTGCGTTTATGTGCCTTGCGGTGGGCATCCTTAGT<br>CACAGCACGCCACTGTTTGTTGGCGTGCTGGTTTACTTCCTGATCGGTACTGCATACA<br>GTGTGGAACTGCCGTTACTGCGTTGGAAAACTAAACCGGCGATGGCGGCGTTTAGCA<br>TGGCAGGTTTGATGGGCCTGACGATTCAACCAGCAGTGTTTTATCACATTCAAAACG<br>CCCTGGGCAAACCGATGGTTTTTTCCAAGACCGTCGCCTTTGCAACGATTTTCTTCAG

	CGTGTTTGCGGCGGTCCTTGGTGCAATTAAAGATGTCCCGGACGTTGAGGGTGATAC<br>TGCGTTTGGTAACCGTACGTTTAGCGTGCGTTATGGTCAGGAGAAAGTTTTCAGTGTT<br>TGTCTGAACATTCTCCTCTTAGCCTACGGCTTTGCAGTCGTGGTCGGCGCGAGTAGCA<br>GCTTCCTGTTATGCAAAATCGTGAGCGTTATGGGCCATACGACGTTAGCGTCGTTGTT<br>ACTTCTGCGCGCCAAAAGTACGAATCCAAAAGATCCGGAGAGCACCCAGAGTTTCT<br>ACATGTTTCTGTTCAAACTTCTCTATGCAGAGTATGTC<br>CTGATCCACTTCATGCGTTAA
PS (*Ammi majus*)	ATGAAAATGCTTGAACAGAACCCGCAGTACCTGTACTTTTTTTCCTTGTTTCTGGTTA<br>CCATCTTTCTGTATAAATGGCTGACTCTCAAGAAAACTCCGCTGAAAAATTTACCG<br>CCGAGCCCGCCGCAATACCCGATTATCGGTAATCTGCACCAGATTGGTCCGGACCC<br>GCAAGCATCCTTACGTGATTTGGCCCAGAAGTATGGTCCGCTGATGTTCCTGAAATT<br>TGGCACGGTGCCGGTTCTGGTTGTTTCGTCCGCGGACGCGGCACGCGAGGCGCTGA<br>AAACCCACGATCTGGTGTTTGCGGATCGTCCGTACAGTAGCGTTGCAAATAAAATT<br>TTTTATAACGGCAAAGATATGGTTTTTGCGCGCTATACGGAATACTGGCGTCAGGTT<br>AAGAGTATTTGCGTTACCCAGCTGTTGAGTAATAAACGCGTTAACAGCTTTCATTAT<br>GTGCGTGAGGAAGAAGTTGATCTCCTGGTGCAGAATCTGGAGAATAGTCATAGCA<br>AAGTGGCGAACTTGACGGAATTACTGATTGAAGTTACGGGCAATGTCGTGTGTCGC<br>GTTAGTGTGGGCAGTGGCGATAAAGTCGATAGTTATAAAATTCTTATTCTGGAGAT<br>CATGGACATGCTGGGTTATAGCCGCAGCATCGAAGACTTTTTCCCATTACTGGGTTG<br>GGTTGATTGGCTGACCGGTCTGCGCGGTAAAGTGGCGGAAGCGGCGAAAGGTGTG<br>GATACGTTCCTTGAGGGCGTTTTGAAGGAACACCTGAGCACGACCGGCTCCAAATA<br>CAATGATTTCGTTAGCATTTTACTGGAAATTCAGGAAGCAGATGCGGGCAGCAGTA<br>TGGATAACGAGTGCATTAAAAGCCTGATTTGGGACATGCTGGGCGCGGGTACGGA<br>AACCATTAGCACGGCGCTTGAATGGACGCTGGCCGCACTGATCAAGAATCCAGAC<br>GCGATGTTTAAACTCCAGAACGAAGTGCGCGAAATCGGCAAAGGCAAAAGCAAA<br>ATCAGCGAGGCGGACCTGGTGAAAATGAATTATCTGCAGGCGGTGATGAAGGAAA<br>GTATGCGCTTGTATTTTACGGCGCCGCTTCTGGTGCCGCGCGAAGCGCGCCAGGAC<br>ATCAAATTTATGGGCTATGACATCAGCAGTGGCACCCAAGTCCTGATTAATGCGTG<br>GGCAATCGCCCGCGATCCGCTGCTGTGGGACAAACCGGAAGAATTTCGCCCGGAA<br>CGTTTTTTGAATTCGCCGATCGACTACAAAGGTTTTCACTATGAATTCCTGCCATTC<br>GGTGCAGGCCGCCGCGGTTGCCCAGGCATTCAGTTCGCAATGTGCATCAACGAACT<br>GGTTGTTGCGAACCTGGTGCACAAATTCAATTTTGAGCTGCCAGATGGTAAACGTC<br>TGGAAGACCTGGATATGACCGCGGCGAGTGGCATTACTCTTCGCAAAAAATCCCCA<br>CTGCTCGTTGTGGCCCGTCCGCACGTGTAA

Table S2. Primers for PCR amplification of the genes of the biosynthetic pathway (forward (FW) and reverse (REV) primers) and sequencing.

Primer name	Primer sequence[1]	Restriction enzyme site
C2′H_pET_FW/ C2′H_pRSF_FW	ACATG*CCATGG*AA**ATG**CCGTCGACCACTCT	NcoI
C2′H_pET_REV/ C2′H_pRSF_S-tag_REV	TTTTTT*CTCGAG*TTCAATGCGCGCGAATG	XhoI
C2′H_pRSF_REV/ C2′H_pRSF_His_REV	AACCC*AAGCTT***TTA**TTCAATGCGCGCGAAT	HindIII
C2′H_pRSF_His_FW	AACGC*GGATCC*G**ATG**CCGTCGACCACTCT	BamHI
C2′H_pRSF_S-tag_FW	AAAAAA*CAT**ATG***CCGTCGACCACTCT	NdeI
*Pc*PT_pETDuet_FW	AAAAAA*CAT**ATG***AGCCAGACCCTGAT	NdeI
*Pc*PT_pETDuet_REV	AAAAA*CTCGAG***TTA**ACGCATGAAGTG	XhoI
*Pc*PT_pRSF/pCDF_FW	AAAAA*GGATCC*G**ATG**AGCCAGA	BamHI
*Pc*PT/Δ48*Pc*PT_pRSF/pCDF/pET28_REV	AAAAA*AAGCTT***TTA**ACGCATGAAGTG	HindIII
*Pc*PT_pET28_FW	AAAAA*CCATGG*AA**ATG**AGCCAGACCCTGAT	NcoI
Δ48*Pc*PT_pRSF/pCDF_FW	AAAAA*GGATCC*G**ATG**GCGTGCCACAAAAACGTTGATT	BamHI
Δ48*Pc*PT_pET28_FW	AAAAA*CCATGG*AA**ATG**GCGTGCCACAAAAACGTTGATT	NcoI
*Ps*PT_pET28_FW	AAAAA*CCATGG*AA**ATG**GCGCAGACCAT	NcoI
*Ps*PT_pET28_REV	AAAAA*AAGCTT*ACGCATGAAGTGGATCA	HindIII
*Ps*PT_pRSF_FW	AAAAA*GGATCC*A**ATG**GCGCAGACCATTATG	BamHI
*Ps*PT_pRSF_REV	AAAAA*AAGCTT***TTA**ACGCATGAAGTGGATCA	HindIII
Δ48*Ps*PT1_FW	AAAAA*CAT**ATG***GCGTGCGATAAAAATTTAGACTCCA	NdeI
Δ48*Ps*PT1_FW	AAAAA*CTCGAG*ACGCATGAAGTGGATCAGG	XhoI
PS_pRSF_FW	AAAAA*GGATCC*A**ATG**AAAATGCTTGAACAGAA	BamHI
PS_pRSF_REV	AAAAA*AAGCTT***TTA**CACGTGCGGACGG	HindIII
PS_pET_FW	AAAAA*CCATGG*AA*ATG*AAAATGCTTGAAC	NcoI
PS_pET_REV	AAAAA*AAGCTT*CACGTGCGGACGGG	HindIII
MA-Δ37PS_FW	AAAAA*CAT**ATGGCG***CCGCCGAGCCCGCCG	NdeI
MA/8RP/28tag/2C3-Δ37PS_REV	AAAAA*CTCGAG*CACGTGCGGACGGGCC	XhoI
8RP-Δ37PS_FW	AAAAA*CAT**ATG***GCTCTGTTATTAGCAGTTTTTCCGCCGAGCCCGCCG	NdeI
28tag-Δ37PS_FW	AAAAA*CAT**ATG***GAATTATCACAAGTTTGTACAAAAAAGGAGGCTGGCGCCGGCACCAATTCAGTGGACTGGATTCAAGAAGGCGATATTACCCCGCCGAGCCCGCCG	NdeI
2C3-Δ37PS_FW	AAAAA*CAT**ATG***GCTAAAAAAACCAGCAGCAAAGGTAAACCGCCGAGCCCGCC	NdeI
GroES_FW	AAAAA*GGATCC*G**ATG**AATATTCGTCCATTGCATGATCGC	BamHI
GroEL_REV	AAAAA*GTCGAC***TTA**CATCATGCCGCCCATGCC	SalI
MS_FW	AAAAA*CAT**ATG***GATATTTTCACCTCCTTAC	NdeI
MS_REV	AAAAA*CTCGAG***CTA**ATGCTTTGTCGGCAC	XhoI
8RP-Δ25MS_FW	AAAAA*CAT**ATG***GCTCTGTTATTAGCAGTTTTTTTTCCTAAACACAAAAGGC	NdeI
Seq_MCS1_FW	GGATCTCGACGCTCTCCCTT	-
Seq_MCS2_FW	GTACACGGCCGCATAATCG	-
Seq_REV	CTAGTTATTGCTCAGCGGT	-

[1] Start and stop codons in **bold**; restriction sites in *italic*; N-terminal modifications are underlined. In order for the sequence to remain in frame one or two bases were occasionally added between the restriction site and the gene start codon.

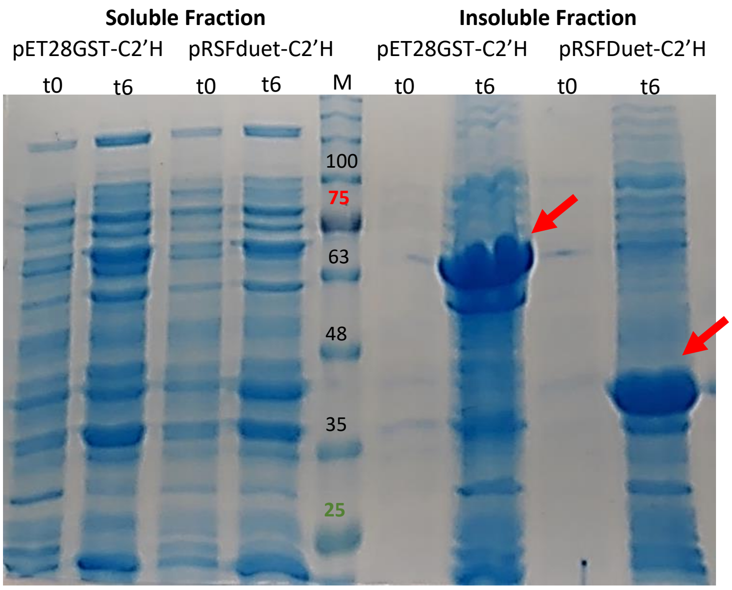

Figure S1. Protein SDS gels showing *p*-coumaroyl-CoA 2′-hydroxylase (C2′H) expression using different plasmids (pRSFDuet-C2′H and pET28GST-C2′H) at time zero (t0) of induction and after 6 h (t6) of induction. C2′H is expected around 40.41 kDa and GST+C2′H at 67.65 kDa. Arrows indicate where it is possible to observe the bands of interest. M: marker (NZYColour Protein Marker II – NZYTech).

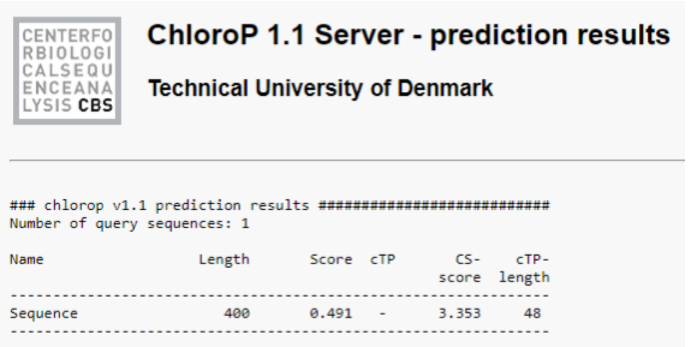

Figure S2. ChloroP results for PT from *Petroselinum crispum*.

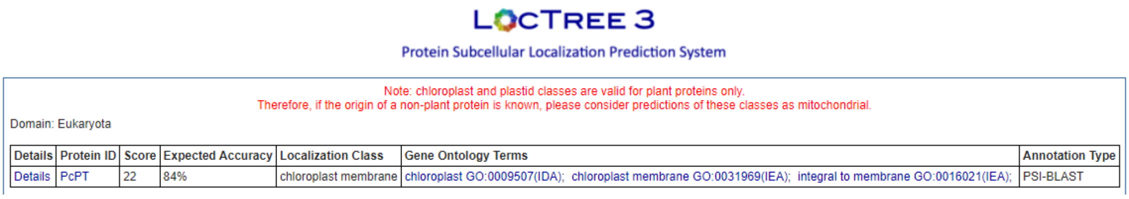

Details	Protein ID	Score	Expected Accuracy	Localization Class	Gene Ontology Terms	Annotation Type
Details	PcPT	22	84%	chloroplast membrane	chloroplast GO:0009507(IDA); chloroplast membrane GO:0031969(IEA); integral to membrane GO:0016021(IEA);	PSI-BLAST

Figure S3. LocTree3 results for PT from *Petroselinum crispum*.

BaCelLo

Balanced Subcellular Localization Predictor

Home
Prediction
Datasets
Proteomes
Info

Results

Predictor chosen: **Plants**

Name of the sequence:	Localization:	Localization Steps:
PcPT	Chloroplast	(Intracellular -> Organelle -> Chloroplast)

Figure S4. BaCelLo results for PT from *Petroselinum crispum*.

iPSORT Prediction

Predicted as: *having a mitochondrial targeting peptide*

Sequence (Type: plant)

```
  1 MSQTL MHSRF SSGFL HHQPE KGFLT LQTQR RHAKT LKGEK EFPSR VVSCH
 51 KNVDS SKNFS SSCEK SKTQE KILAQ TLGAT SDGEA IVQPN NDFEV TWQNT
101 LRRKW DAFSI FSRPY SAICT IIGIS SVSLL PLTSV ADFSP AYFVG LLQAL
151 IPFLC ANIYT SAINQ LVDVD IDKIN KPYLP LVSGE FSMGE GRAIV SALTF
201 TCFAM AIMSH SVPLF VGVLV YFLIG TAYSV EHPLL RWKTK PAMAA FSMAG
251 LMGLT IQPTV FYHIQ NVLGK PMVFS RSVAF ATMFF SIFAA CLGAI KDIPD
301 VEGDR EFGNL TFSVR YGQEK VFSFC LNVLL LAYGS AVVVG ASSSS LLCKT
351 VSVIG HTVLA SLLVL RAKST NPKDP ESTQS FYMFL FKLLY AEYVL IHFMR
```

Values used for reasoning

Node	Answer	View	Substring	Value(s)	Plot
**1. Signal peptide?**	**No**	Average Hydropathy (KYTJ820101)	[6,25]	-0.63 (>= 0.9225? No)	show
**2. Mitochondrial or chloroplast ?**	**Yes**	Average Negative Charge (FAUJ880112)	[1,30]	0.0333333 (< 0.083? Yes)	show
		Indexing: AI1<br>Pattern: 22121222 (ins/del <= 2)	[1,30]	MS-Q-TLMHSRFSSGFLHHQPEKGFLTLQTQR<br>22-2-2220212220220022000222222221<br>22121222	--
**3. Mitochondrial?**	**Yes**	Average Isoelectric Point (ZIMJ680104)	[1,15]	6.18333 (>= 6.21? No)	show
		Indexing: AI2<br>Pattern: 100100110 (ins/del <= 3)	[1,15]	MSQTLM-HS-RFSSGFL<br>000000-00-1000000<br>100100110	--

* This color means "not used".

Figure S5. iPSORT results for PT from *Petroselinum crispum*.

PcPT
Prediction: Other

Protein type	Other	Signal peptide	Mitochondrial transfer peptide	Chloroplast transfer peptide	Thylakoid luminal transfer peptide
Likelihood	0.3199	0.0002	0.2763	0.3189	0.0848

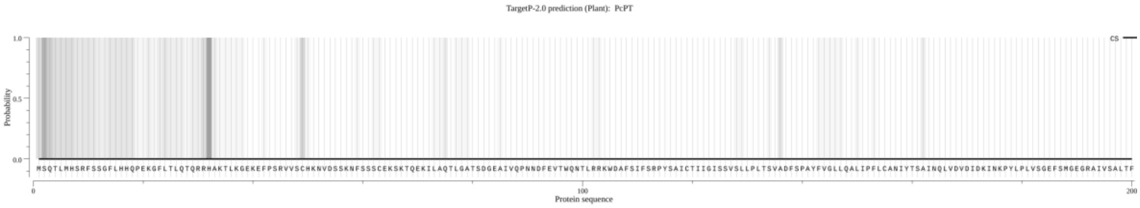

Figure S6. TargetP-2.0 results for PT from *Petroselinum crispum*.

PcPT

Prediction: Other

Protein type	Signal Peptide (Sec/SPI)	Other
Likelihood	0.0003	0.9997

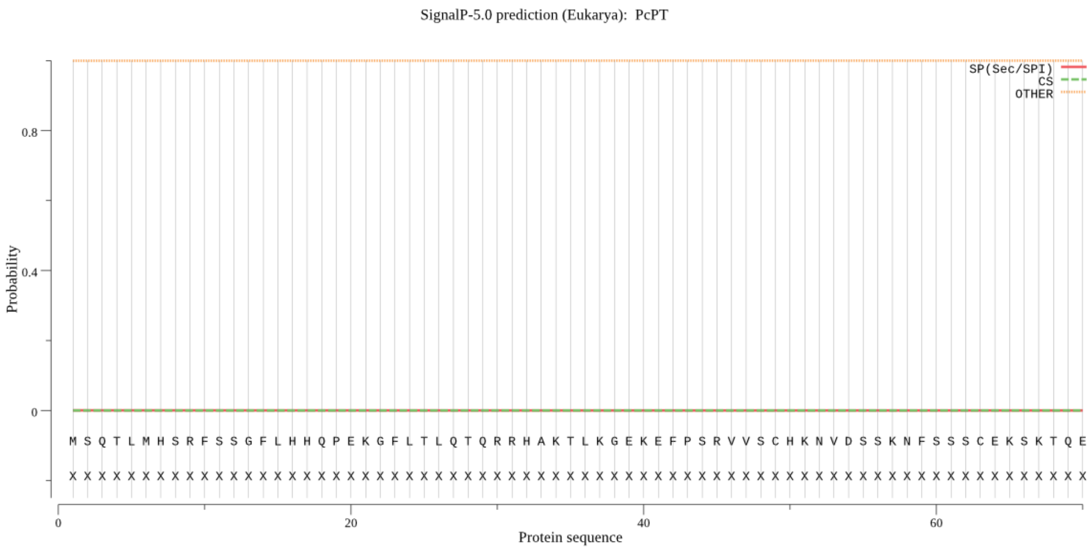

Figure S7. SignalP-5.0 results for PT from *Petroselinum crispum*.

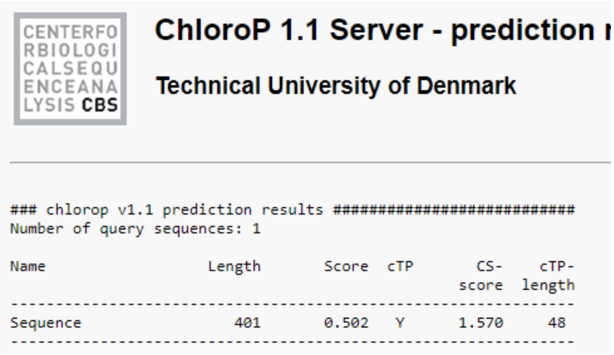

chlorop v1.1 prediction results
Number of query sequences: 1

Name	Length	Score	cTP	CS-score	cTP-length
Sequence	401	0.502	Y	1.570	48

Figure S8. ChloroP results for PT1 from *Pastinaca sativa*.

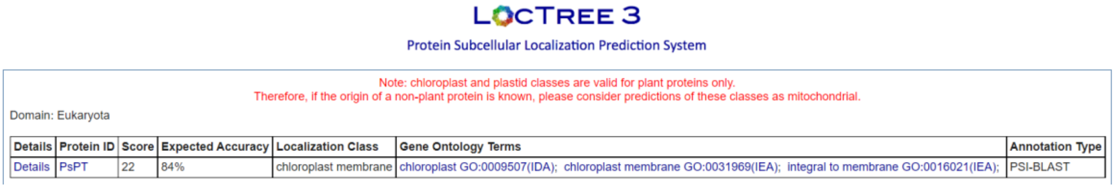

Note: chloroplast and plastid classes are valid for plant proteins only.
Therefore, if the origin of a non-plant protein is known, please consider predictions of these classes as mitochondrial.

Domain: Eukaryota

Details	Protein ID	Score	Expected Accuracy	Localization Class	Gene Ontology Terms	Annotation Type
Details	PsPT	22	84%	chloroplast membrane	chloroplast GO:0009507(IDA); chloroplast membrane GO:0031969(IEA); integral to membrane GO:0016021(IEA);	PSI-BLAST

Figure S9. LocTree3 results for PT1 from *Pastinaca sativa*.

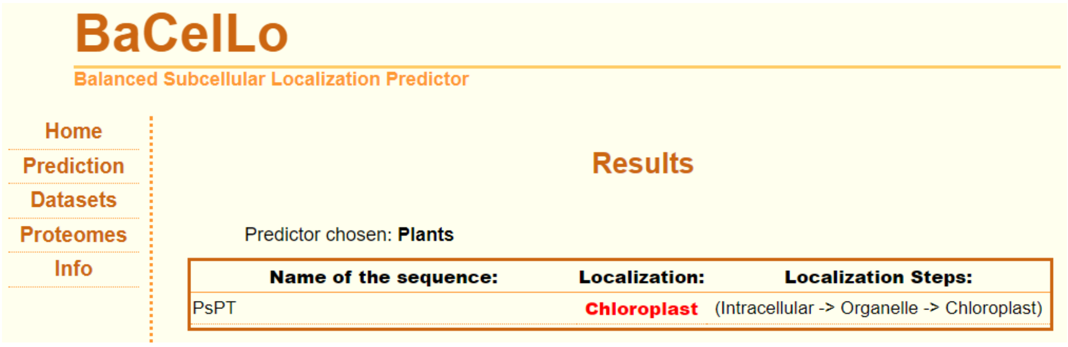

BaCelLo

Balanced Subcellular Localization Predictor

Home
Prediction
Datasets
Proteomes
Info

Results

Predictor chosen: **Plants**

Name of the sequence:	Localization:	Localization Steps:
PsPT	Chloroplast	(Intracellular -> Organelle -> Chloroplast)

Figure S10. BaCelLo results for PT1 from *Pastinaca sativa*.

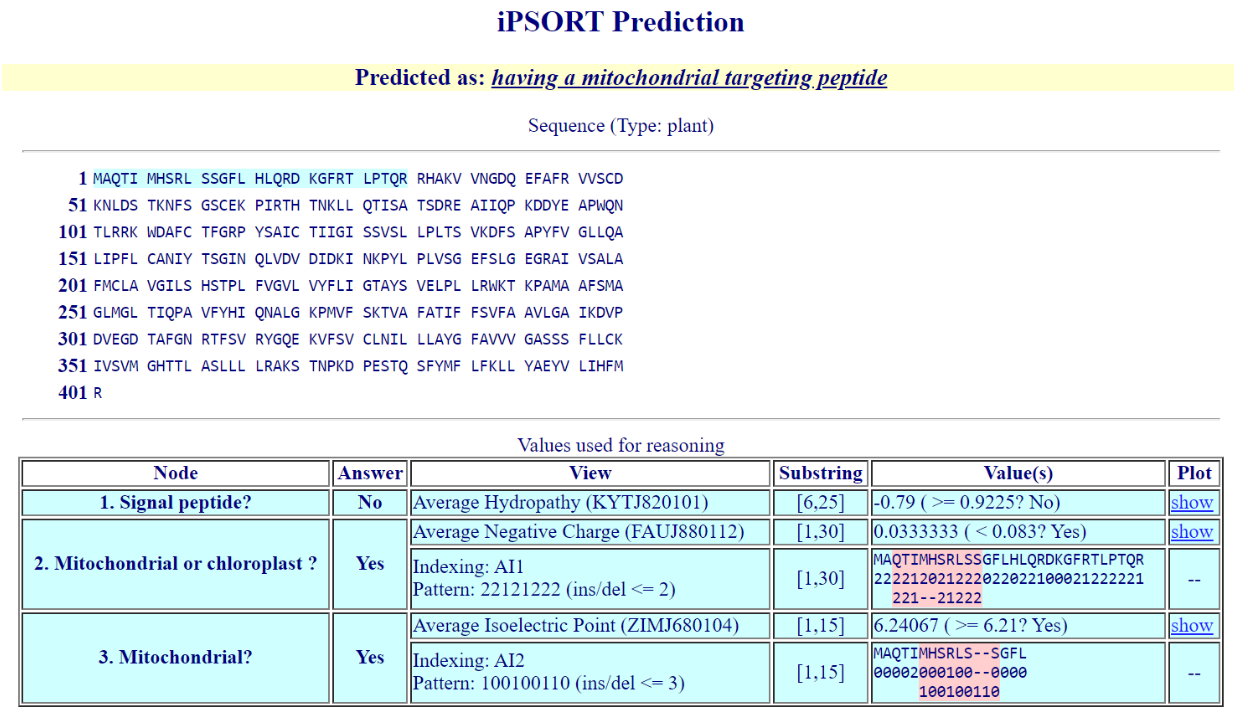

iPSORT Prediction

Predicted as: *having a mitochondrial targeting peptide*

Sequence (Type: plant)

```
  1 MAQTI MHSRL SSGFL HLQRD KGFRT LPTQR RHAKV VNGDQ EFAFR VVSCD
 51 KNLDS TKNFS GSCEK PIRTH TNKLL QTISA TSDRE AIIQP KDDYE APWQN
101 TLRRK WDAFC TFGRP YSAIC TIIGI SSVSL LPLTS VKDFS APYFV GLLQA
151 LIPFL CANIY TSGIN QLVDV DIDKI NKPYL PLVSG EFSLG EGRAI VSALA
201 FMCLA VGILS HSTPL FVGVL VYFLI GTAYS VELPL LRWKT KPAMA AFSMA
251 GLMGL TIQPA VFYHI QNALG KPMVF SKTVA FATIF FSVFA AVLGA IKDVP
301 DVEGD TAFGN RTFSV RYGQE KVFSV CLNIL LLAYG FAVVV GASSS FLLCK
351 IVSVM GHTTL ASLLL LRAKS TNPKD PESTQ SFYMF LFKLL YAEYV LIHFM
401 R
```

Values used for reasoning

Node	Answer	View	Substring	Value(s)	Plot
1. Signal peptide?	No	Average Hydropathy (KYTJ820101)	[6,25]	-0.79 (>= 0.9225? No)	show
2. Mitochondrial or chloroplast ?	Yes	Average Negative Charge (FAUJ880112)	[1,30]	0.0333333 (< 0.083? Yes)	show
		Indexing: AI1<br>Pattern: 22121222 (ins/del <= 2)	[1,30]	MAQTIMHSRLSSGFLHLQRDKGFRTLPTQR<br>222212021222022022100021222221<br>221--21222	--
3. Mitochondrial?	Yes	Average Isoelectric Point (ZIMJ680104)	[1,15]	6.24067 (>= 6.21? Yes)	show
		Indexing: AI2<br>Pattern: 100100110 (ins/del <= 3)	[1,15]	MAQTIMHSRLS--SGFL<br>00002000100--0000<br>100100110	--

Figure S11. iPSORT results for PT1 from *Pastinaca sativa*.

Sequence
Prediction: Mitochondrial transfer peptide

CS pos: 32-33. RRH-AK. Pr: 0.7081

Protein type	Other	Signal peptide	Mitochondrial transfer peptide	Chloroplast transfer peptide	Thylakoid luminal transfer peptide
**Likelihood**	0.3569	0.001	0.5376	0.0989	0.0056

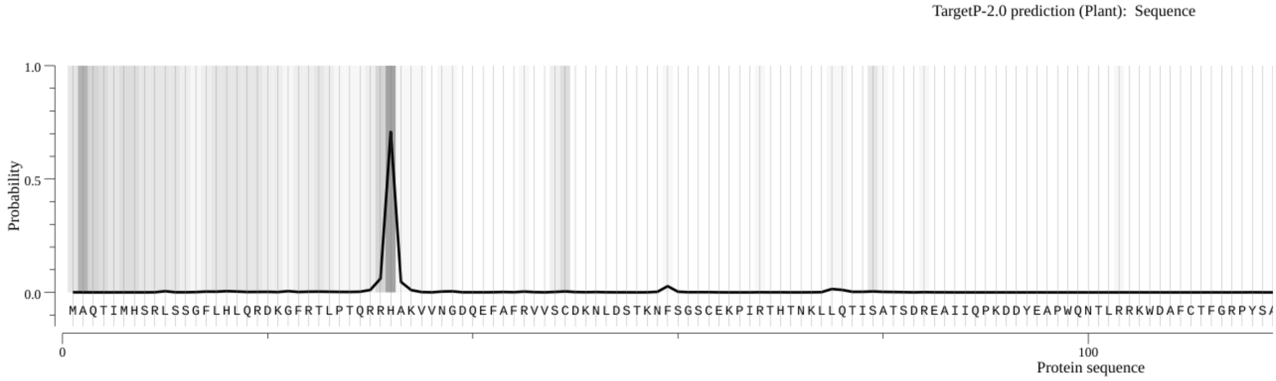

Figure S12. TargetP results for PT1 from *Pastinaca sativa*.

Prediction: Other

Protein type	Signal Peptide (Sec/SPI)	Other
**Likelihood**	0.0006	0.9994

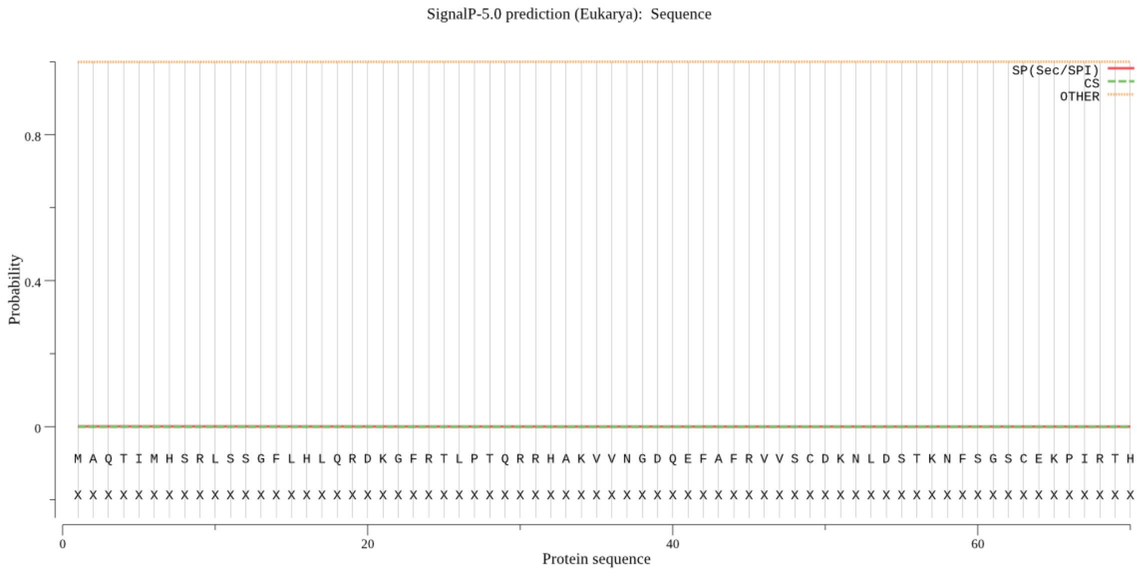

Figure S13. SignalP-5.0 results for PT1 from *Pastinaca sativa*.

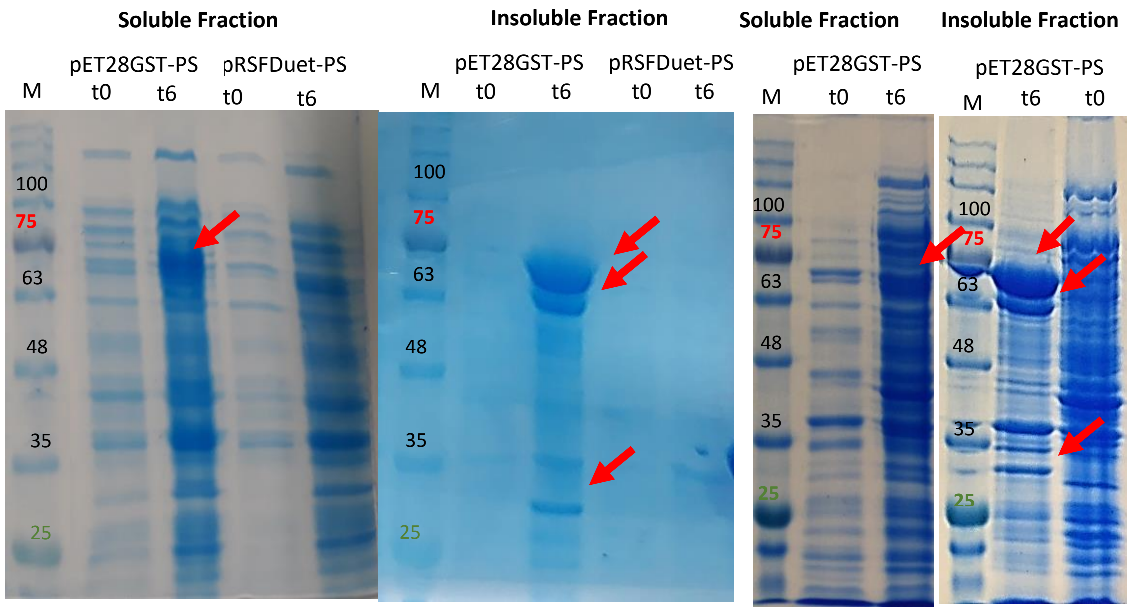

Figure S14. Protein SDS gels showing psoralen synthase (PS) expression using different plasmids (pRSFDuet-PS and pET28GST-PS) at time zero (t0) of induction and after 6 h (t6) of induction. PS is expected around 55.9 kDa and GST+PS at 82.08 kDa. Arrows indicate where it is possible to observe the bands of interest. M: marker (NZYColour Protein Marker II – NZYTech).

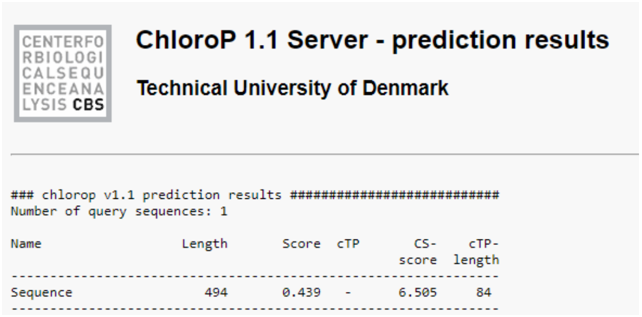

CENTERFO RBIOLOGI CALSEQU ENCEANA LYSIS CBS

ChloroP 1.1 Server - prediction results

Technical University of Denmark

```
### chlorop v1.1 prediction results ##########################
Number of query sequences: 1

Name                 Length       Score  cTP       CS-    cTP-
                                                  score  length
----------------------------------------------------------------
Sequence                494       0.439   -       6.505     84
----------------------------------------------------------------
```

Figure S15. ChloroP results for PS from *Ammi majus*.

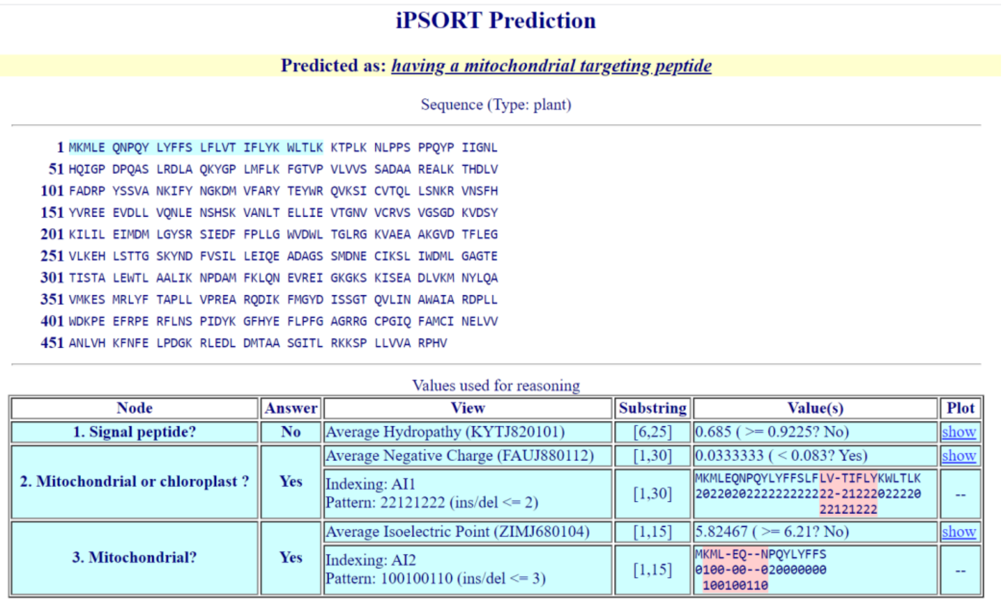

iPSORT Prediction

Predicted as: ***having a mitochondrial targeting peptide***

Sequence (Type: plant)

```
  1 MKMLE QNPQY LYFFS LFLVT IFLYK WLTLK KTPLK NLPPS PPQYP IIGNL
 51 HQIGP DPQAS LRDLA QKYGP LMFLK FGTVP VLVVS SADAA REALK THDLV
101 FADRP YSSVA NKIFY NGKDM VFARY TEYWR QVKSI CVTQL LSNKR VNSFH
151 YVREE EVDLL VQNLE NSHSK VANLT ELLIE VTGNV VCRVS VGSGD KVDSY
201 KILIL EIMDM LGYSR SIEDF FPLLG WVDWL TGLRG KVAEA AKGVD TFLEG
251 VLKEH LSTTG SKYND FVSIL LEIQE ADAGS SMDNE CIKSL IWDML GAGTE
301 TISTA LEWTL AALIK NPDAM FKLQN EVREI GKGKS KISEA DLVKM NYLQA
351 VMKES MRLYF TAPLL VPREA RQDIK FMGYD ISSGT QVLIN AWAIA RDPLL
401 WDKPE EFRPE RFLNS PIDYK GFHYE FLPFG AGRRG CPGIQ FAMCI NELVV
451 ANLVH KFNFE LPDGK RLEDL DMTAA SGITL RKKSP LLVVA RPHV
```

Values used for reasoning

Node	Answer	View	Substring	Value(s)	Plot
1. Signal peptide?	No	Average Hydropathy (KYTJ820101)	[6,25]	0.685 (>= 0.9225? No)	show
2. Mitochondrial or chloroplast ?	Yes	Average Negative Charge (FAUJ880112)	[1,30]	0.0333333 (< 0.083? Yes)	show
		Indexing: AI1<br>Pattern: 22121222 (ins/del <= 2)	[1,30]	MKMLEQNPQYLYFFSLFLV-TIFLYKWLTLK<br>2022020222222222222-21222022220<br>22121222	--
3. Mitochondrial?	Yes	Average Isoelectric Point (ZIMJ680104)	[1,15]	5.82467 (>= 6.21? No)	show
		Indexing: AI2<br>Pattern: 100100110 (ins/del <= 3)	[1,15]	MKML-EQ--NPQYLYFFS<br>0100-00--020000000<br>100100110	--

Figure S16. iPSORT results for PS from *Ammi majus.*

TMHMM result

```
# PS Length: 494
# PS Number of predicted TMHs:  1
# PS Exp number of AAs in TMHs: 21.9297699999999
# PS Exp number, first 60 AAs:  19.06224
# PS Total prob of N-in:        0.80603
# PS POSSIBLE N-term signal sequence
PS      TMHMM2.0        inside       1    11
PS      TMHMM2.0        TMhelix     12    29
PS      TMHMM2.0        outside     30   494
```

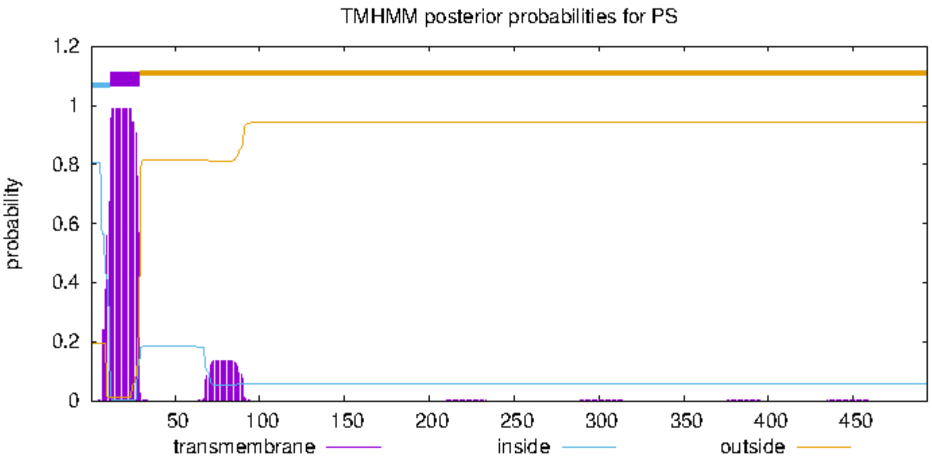

Figure S17. TMHMM-2.0 results for PS from *Ammi majus.*

LocTree 3

Protein Subcellular Localization Prediction System

Domain: Eukaryota

Details	Protein ID	Score	Expected Accuracy	Localization Class	Gene Ontology Terms	Annotation Type
Details	PS	25	84%	endoplasmic reticulum	endoplasmic reticulum GO:0005783(IEA);	PSI-BLAST

Figure S18. LocTree3 results for PS from *Ammi majus.*

BaCelLo

Balanced Subcellular Localization Predictor

Home
Prediction
Datasets
Proteomes
Info

Results

Predictor chosen: **Plants**

Name of the sequence:	Localization:	Localization Steps:
PS	Chloroplast	(Intracellular -> Organelle -> Chloroplast)

Figure S19. BaCelLo results for PS from *Ammi majus.*

PS
Prediction: Other

Protein type	Other	Signal peptide	Mitochondrial transfer peptide	Chloroplast transfer peptide	Thylakoid luminal transfer peptide
**Likelihood**	0.8774	0.0955	0.0187	0.006	0.0024

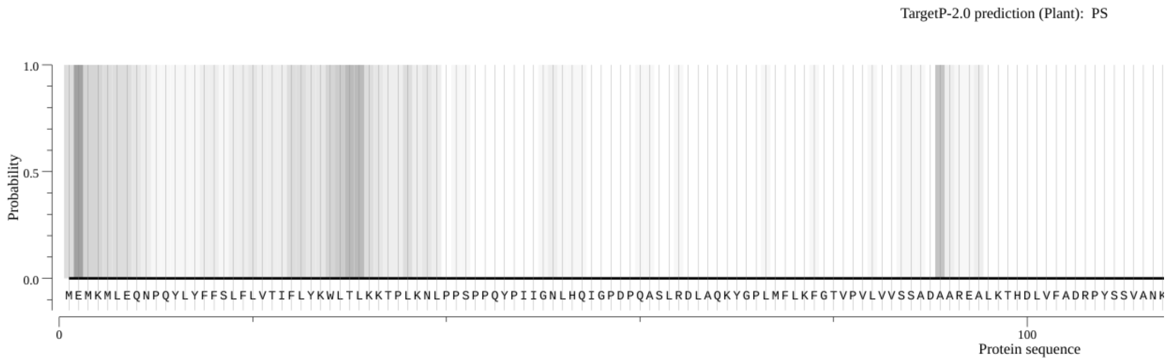

Figure S20. TargetP-2.0 results for PS from *Ammi majus.*

Prediction: Other

**Protein type**	**Signal Peptide (Sec/SPI)**	**Other**
**Likelihood**	0.006	0.994

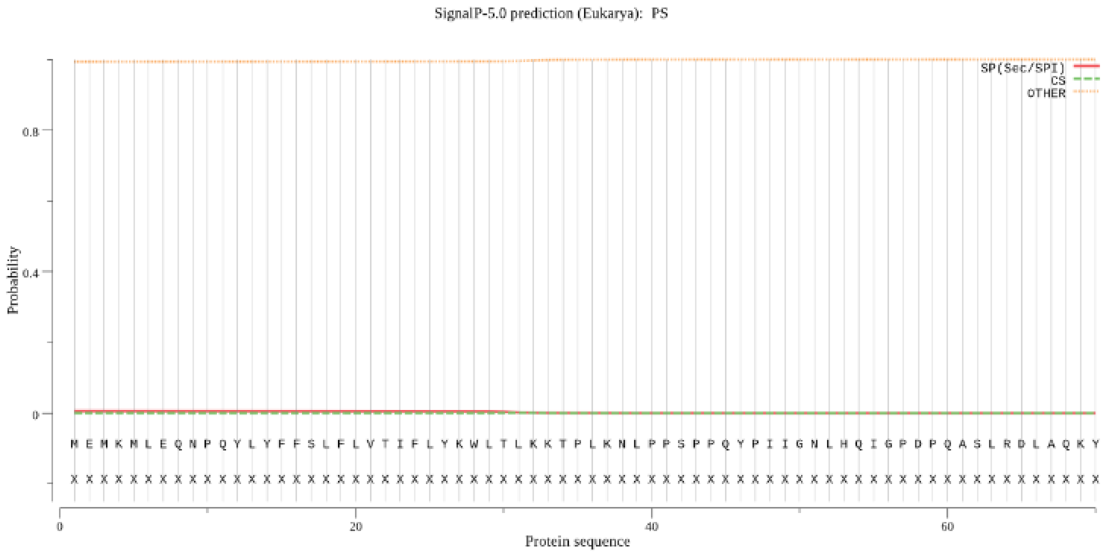

Figure S21. SignalP-5.0 results for PS from *Ammi majus.*

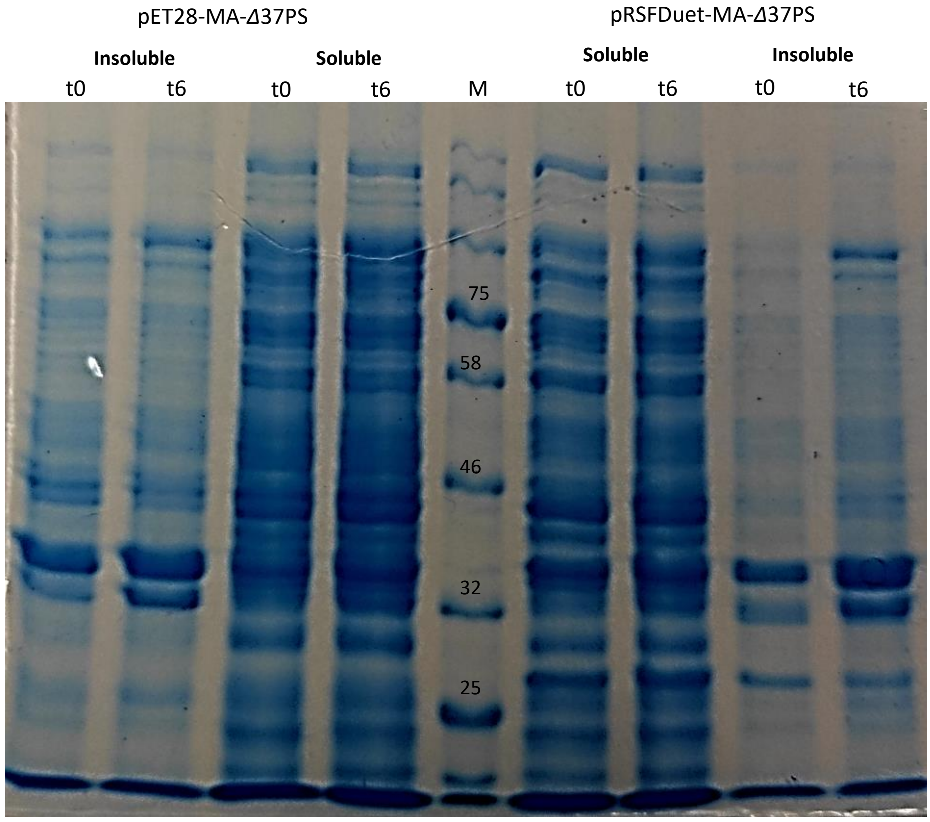

Figure S22. Protein SDS gels showing *Δ*37 aa psoralen synthase (PS) expression with MA N-terminal using different plasmids (pRSFDuet-MA-*Δ*37PS and pET28-MA-*Δ*37PS) at time zero (t0) of induction and after 6 h (t6) of induction. PS is expected around 52-54 kDa. M: marker (Blue Prestained Protein Standard, Broad Range - NEB).

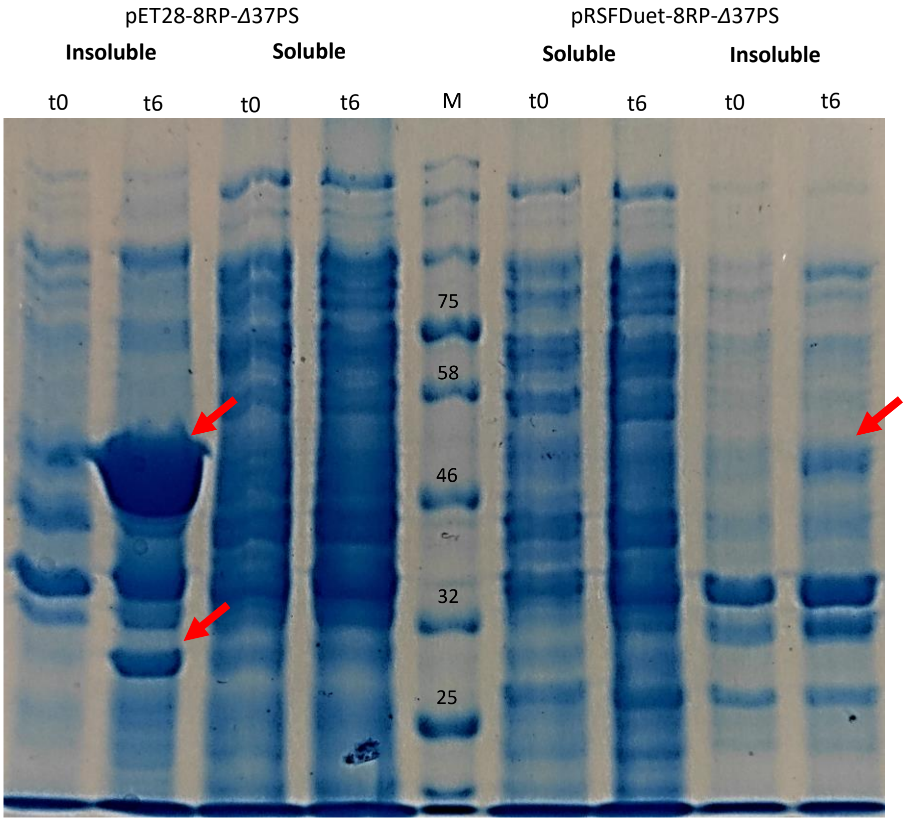

Figure S23. Protein SDS gels showing *Δ*37 aa psoralen synthase (PS) expression with 8RP N-terminal using different plasmids (pRSFDuet-8RP-*Δ*37PS and pET28-8RP-*Δ*37PS) at time zero (t0) of induction and after 6 h (t6) of induction. PS is expected around 53-55 kDa. Arrows indicate where it is possible to observe the bands of interest. M: marker (Blue Prestained Protein Standard, Broad Range - NEB).

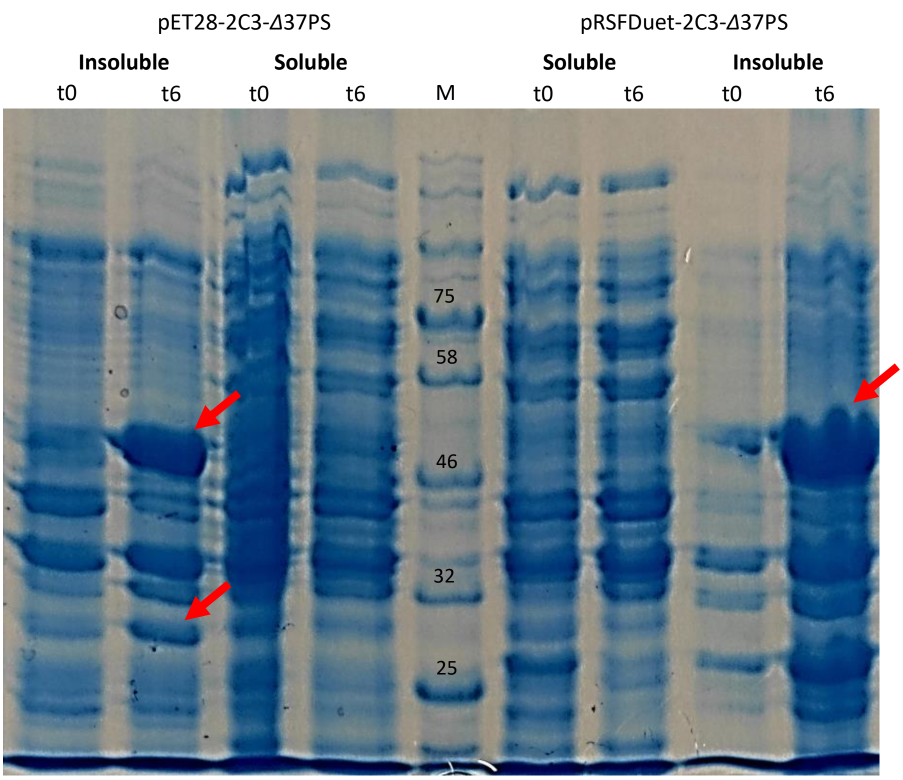

Figure S24. Protein SDS gels showing *Δ*37 aa psoralen synthase (PS) expression with 2C3 N-terminal using different plasmids (pRSFDuet-2C3-*Δ*37PS and pET28-2C3-*Δ*37PS) at time zero (t0) of induction and after 6 h (t6) of induction. PS is expected around 53-55 kDa. Arrows indicate where it is possible to observe the bands of interest. M: marker (Blue Prestained Protein Standard, Broad Range - NEB).

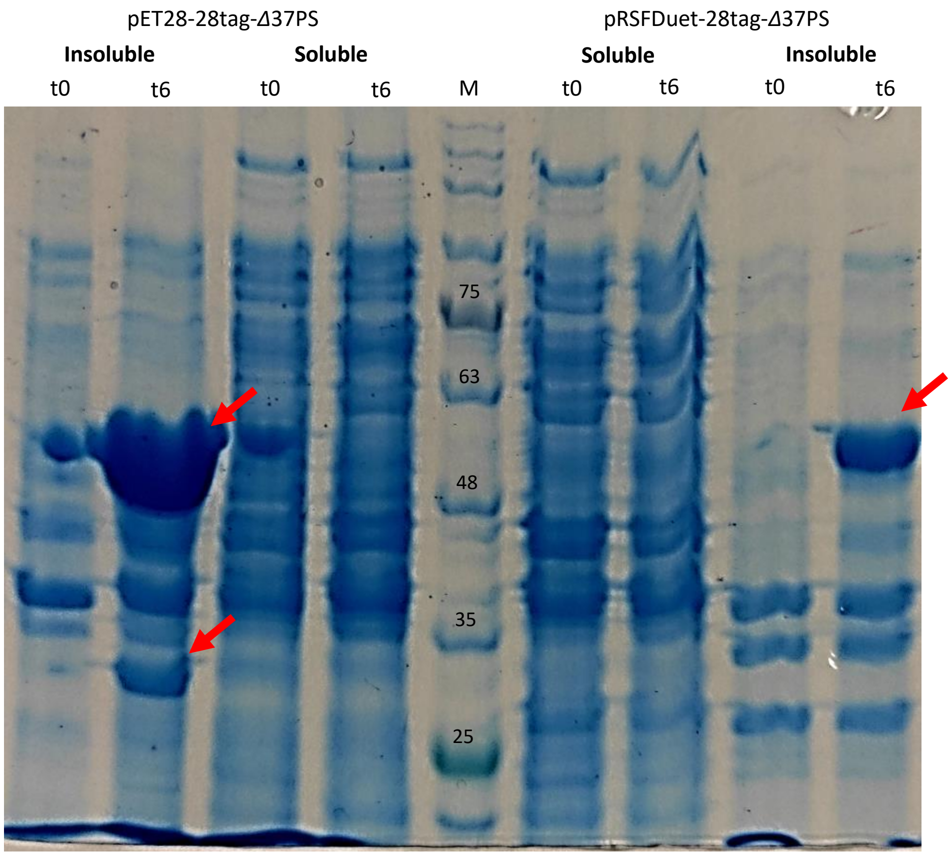

Figure S25. Protein SDS gels showing *Δ*37 aa psoralen synthase (PS) expression with 28tag N-terminal using different plasmids (pRSFDuet-28tag-*Δ*37PS and pET28-28tag-*Δ*37PS) at time zero (t0) of induction and after 6 h (t6) of induction. PS is expected around 55-57 kDa. Arrows indicate where it is possible to observe the bands of interest. M: marker (NZYColour Protein Marker II – NZYTech).

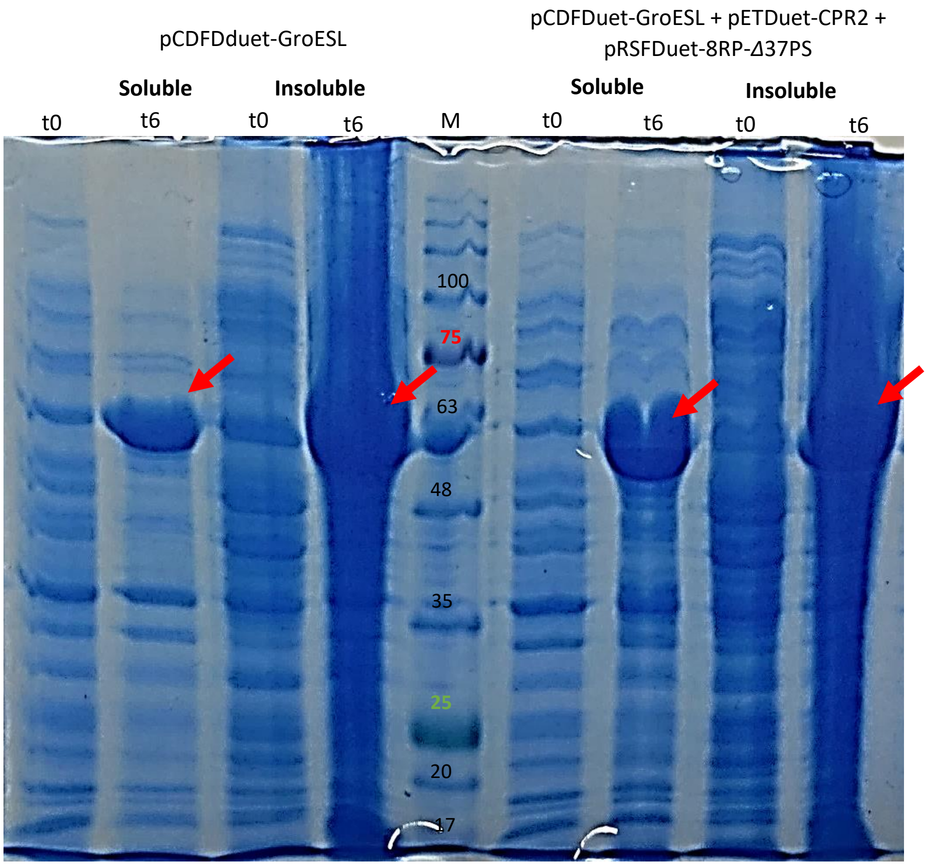

Figure S26. Protein SDS gels showing GroESL expression alone and when combined with cytochrome P450 reductase (CPR2) and 8RP-Δ37PS at time zero (t0) of induction and after 6 h (t6) of induction. GroEL, GroES, CPR2 and 8RP-Δ37PS are expected at round 57, 10, 79 and 55 kDa, respectively. Arrows indicate where it is possible to observe the bands of interest. M: marker (NZYColour Protein Marker II – NZYTech).

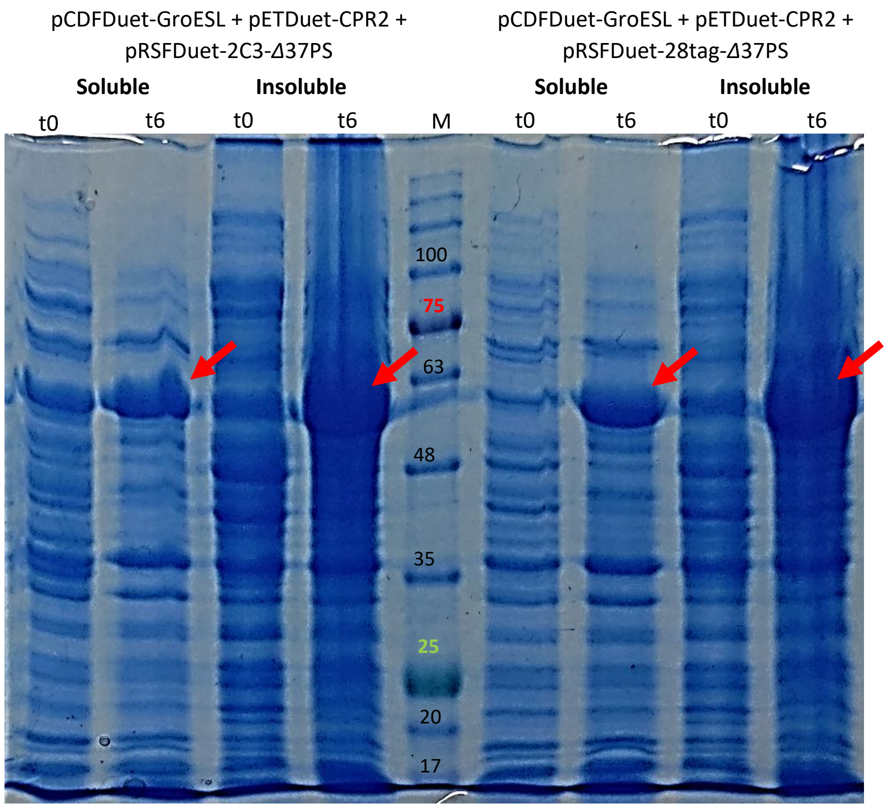

Figure S27. Protein SDS gels showing GroESL expression combined with cytochrome P450 reductase (CPR2) and 2C3-Δ37PS or 28tag-Δ37PS at time zero (t0) of induction and after 6 h (t6) of induction. GroEL, GroES, CPR2, 2C3-Δ37PS and 28tag-Δ37PS are expected at round 57, 10, 79, 55 and 56 kDa, respectively. Arrows indicate where it is possible to observe the bands of interest. M: marker (NZYColour Protein Marker II – NZYTech).

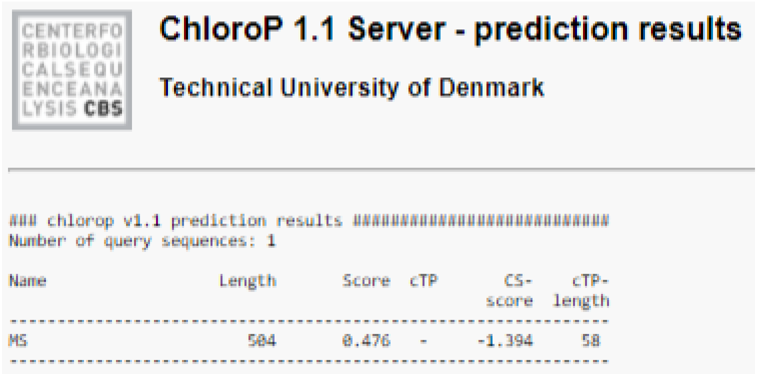

CENTERFORBIOLOGICALSEQUENCEANALYSIS CBS

ChloroP 1.1 Server - prediction results

Technical University of Denmark

chlorop v1.1 prediction results
Number of query sequences: 1

Name	Length	Score	cTP	CS-score	cTP-length
MS	504	0.476	-	-1.394	58

Figure S28. ChloroP results for MS from *Ficus carica.*

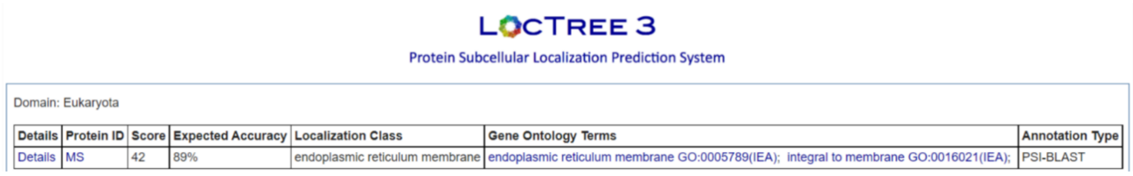

LocTree 3
Protein Subcellular Localization Prediction System

Domain: Eukaryota

Details	Protein ID	Score	Expected Accuracy	Localization Class	Gene Ontology Terms	Annotation Type
Details	MS	42	89%	endoplasmic reticulum membrane	endoplasmic reticulum membrane GO:0005789(IEA); integral to membrane GO:0016021(IEA);	PSI-BLAST

Figure S29. LocTree3 results for MS from *Ficus carica.*

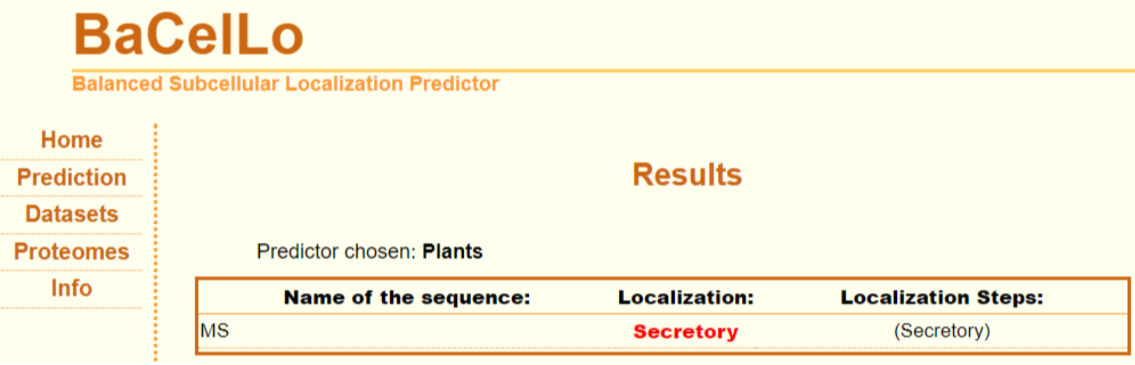

BaCelLo
Balanced Subcellular Localization Predictor

Home
Prediction
Datasets
Proteomes
Info

Results

Predictor chosen: **Plants**

Name of the sequence:	Localization:	Localization Steps:
MS	Secretory	(Secretory)

Figure S30. BaCelLo results for MS from *Ficus carica.*

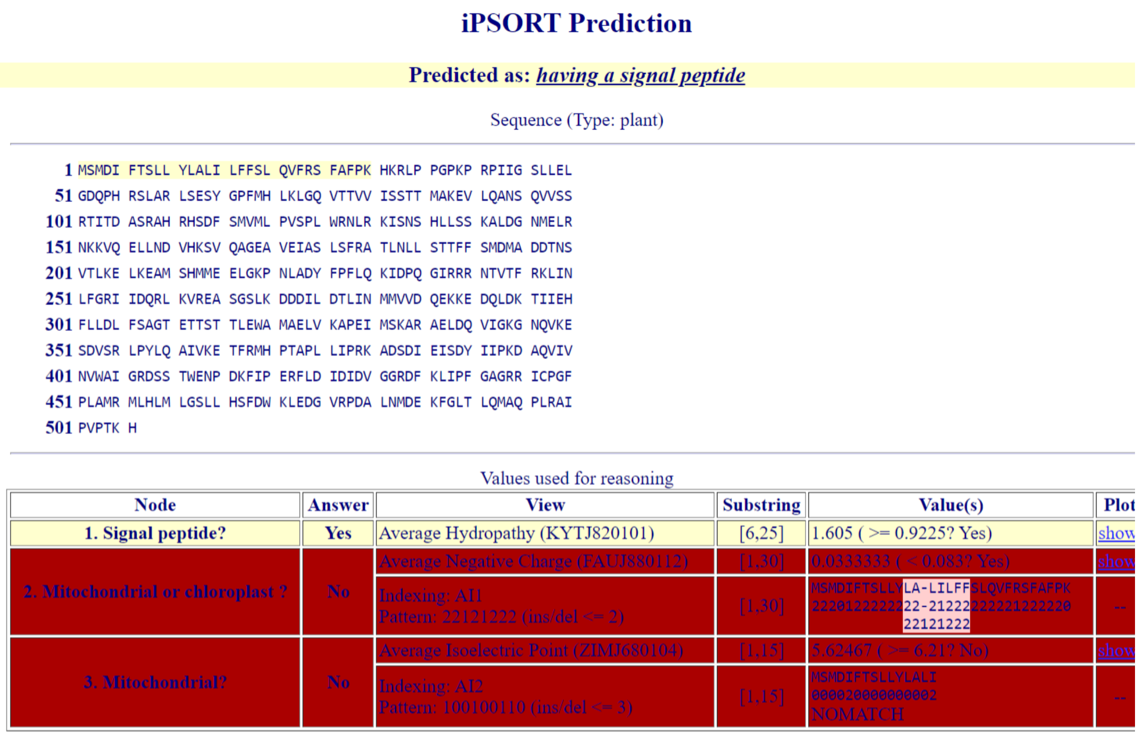

iPSORT Prediction

Predicted as: having a signal peptide

Sequence (Type: plant)

```
  1 MSMDI FTSLL YLALI LFFSL QVFRS FAFPK HKRLP PGPKP RPIIG SLLEL
 51 GDQPH RSLAR LSESY GPFMH LKLGQ VTTVV ISSTT MAKEV LQANS QVVSS
101 RTITD ASRAH RHSDF SMVML PVSPL WRNLR KISNS HLLSS KALDG NMELR
151 NKKVQ ELLND VHKSV QAGEA VEIAS LSFRA TLNLL STTFF SMDMA DDTNS
201 VTLKE LKEAM SHMME ELGKP NLADY FPFLQ KIDPQ GIRRR NTVTF RKLIN
251 LFGRI IDQRL KVREA SGSLK DDDIL DTLIN MMVVD QEKKE DQLDK TIIEH
301 FLLDL FSAGT ETTST TLEWA MAELV KAPEI MSKAR AELDQ VIGKG NQVKE
351 SDVSR LPYLQ AIVKE TFRMH PTAPL LIPRK ADSDI EISDY IIPKD AQVIV
401 NVWAI GRDSS TWENP DKFIP ERFLD IDIDV GGRDF KLIPF GAGRR ICPGF
451 PLAMR MLHLM LGSLL HSFDW KLEDG VRPDA LNMDE KFGLT LQMAQ PLRAI
501 PVPTK H
```

Values used for reasoning

Node	Answer	View	Substring	Value(s)	Plot
1. Signal peptide?	Yes	Average Hydropathy (KYTJ820101)	[6,25]	1.605 (>= 0.9225? Yes)	show
2. Mitochondrial or chloroplast ?	No	Average Negative Charge (FAUJ880112)	[1,30]	0.0333333 (< 0.083? Yes)	show
		Indexing: AI1<br>Pattern: 22121222 (ins/del <= 2)	[1,30]	MSMDIFTSLLYLA-LILFFSLQVFRSFAFPK<br>2220122222222-2122222221222220<br>22121222	--
3. Mitochondrial?	No	Average Isoelectric Point (ZIMJ680104)	[1,15]	5.62467 (>= 6.21? No)	show
		Indexing: AI2<br>Pattern: 100100110 (ins/del <= 3)	[1,15]	MSMDIFTSLLYLALI<br>000020000000002<br>NOMATCH	--

Figure S31. iPSORT results for MS from *Ficus carica.*

MS
Prediction: Signal peptide

CS pos: 25-26. SFA-FP. Pr: 0.9070

Protein type	Other	Signal peptide	Mitochondrial transfer peptide	Chloroplast transfer peptide	Thylakoid luminal transfer peptide
**Likelihood**	0.0723	0.9225	0.0027	0.0013	0.0011

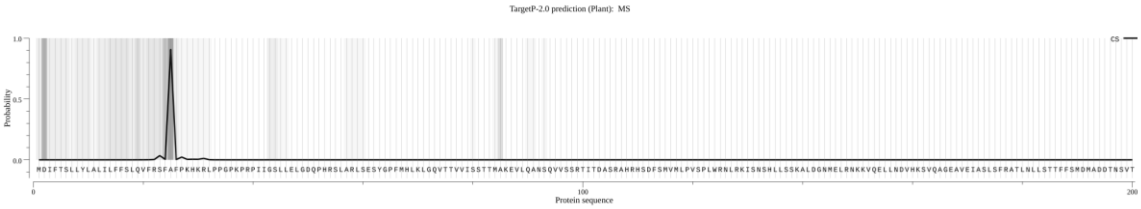

Figure S32. TargetP-2.0 results for MS from *Ficus carica.*

Prediction: Signal peptide (Sec/SPI)

Cleavage site between pos. 25 and 26: SFA-FP. Probability: 0.535[illegible]

Protein type	Signal Peptide (Sec/SPI)	Other
**Likelihood**	0.6097	0.3903

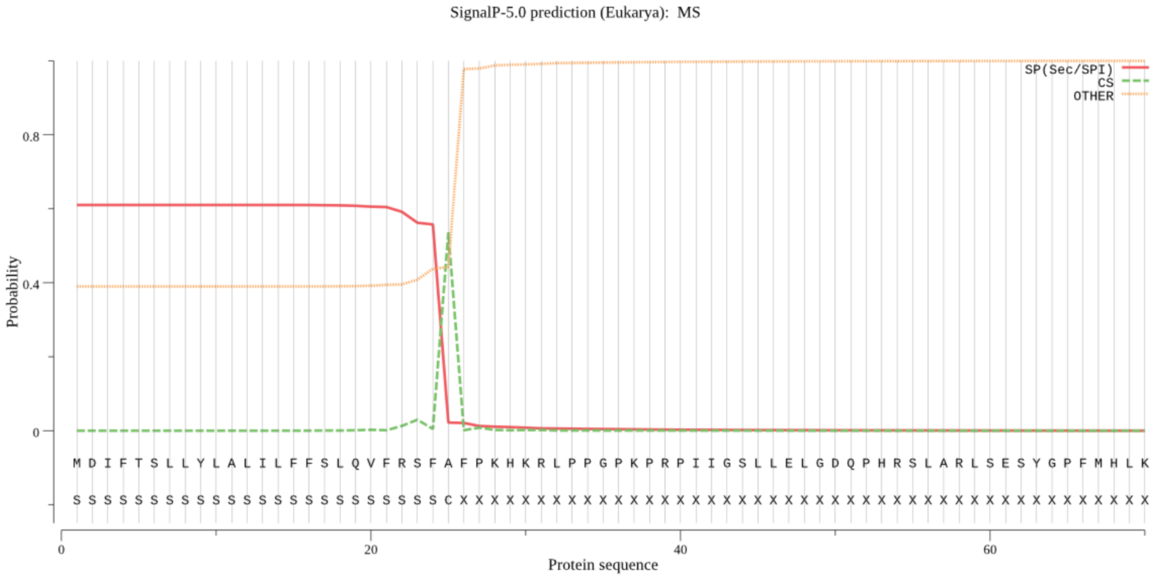

Figure S33. SignalP-5.0 results for MS from *Ficus carica.*

TMHMM result

```
# MS Length: 504
# MS Number of predicted TMHs:  1
# MS Exp number of AAs in TMHs: 19.0928099999999
# MS Exp number, first 60 AAs:  19.04846
# MS Total prob of N-in:        0.95858
# MS POSSIBLE N-term signal sequence
MS      TMHMM2.0        inside       1     6
MS      TMHMM2.0        TMhelix      7    26
MS      TMHMM2.0        outside     27   504
```

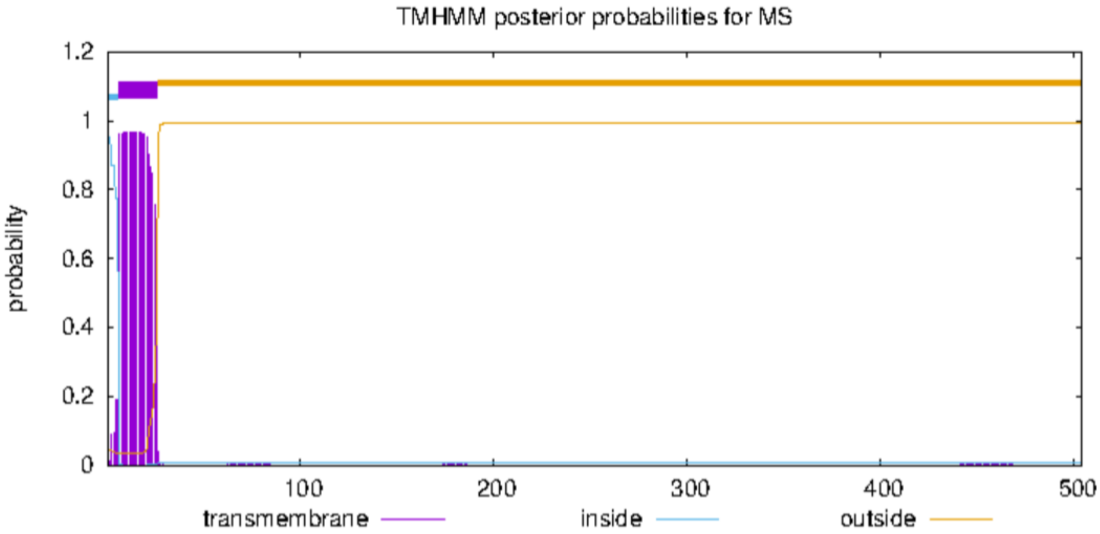

Figure S34. TMHMM-2.0 results for MS from *Ficus carica.*
